# Quantification Assays for Total and Polyglutamine-Expanded Huntingtin Proteins

**DOI:** 10.1371/journal.pone.0096854

**Published:** 2014-05-09

**Authors:** Douglas Macdonald, Michela A. Tessari, Ivette Boogaard, Melanie Smith, Kristiina Pulli, Agnieszka Szynol, Faywell Albertus, Marieke B. A. C. Lamers, Sipke Dijkstra, Daniel Kordt, Wolfgang Reindl, Frank Herrmann, George McAllister, David F. Fischer, Ignacio Munoz-Sanjuan

**Affiliations:** 1 CHDI Management/CHDI Foundation, Los Angeles, California, United States of America; 2 Galapagos B.V., Leiden, The Netherlands; 3 BioFocus, a Charles River company, Saffron Walden, United Kingdom; 4 BioFocus, a Charles River company, Leiden, The Netherlands; 5 Evotec AG, Hamburg, Germany; National Center of Neurology and Psychiatry, Japan

## Abstract

The expansion of a CAG trinucleotide repeat in the huntingtin gene, which produces huntingtin protein with an expanded polyglutamine tract, is the cause of Huntington's disease (HD). Recent studies have reported that RNAi suppression of polyglutamine-expanded huntingtin (mutant HTT) in HD animal models can ameliorate disease phenotypes. A key requirement for such preclinical studies, as well as eventual clinical trials, aimed to reduce mutant HTT exposure is a robust method to measure HTT protein levels in select tissues. We have developed several sensitive and selective assays that measure either total human HTT or polyglutamine-expanded human HTT proteins on the electrochemiluminescence Meso Scale Discovery detection platform with an increased dynamic range over other methods. In addition, we have developed an assay to detect endogenous mouse and rat HTT proteins in pre-clinical models of HD to monitor effects on the wild type protein of both allele selective and non-selective interventions. We demonstrate the application of these assays to measure HTT protein in several HD *in vitro* cellular and *in vivo* animal model systems as well as in HD patient biosamples. Furthermore, we used purified recombinant HTT proteins as standards to quantitate the absolute amount of HTT protein in such biosamples.

## Introduction

The measurement of disease-causing mutant and/or misfolded proteins is essential for the successful development of disease-modifying therapies that target such pathogenic or pathologic proteins. Development of assays to detect these types of proteins is dependent on the availability of selective antibodies as well as a sensitive and robust platform for detection. To this end, we have characterized a set of novel antibodies and employed a unique assay platform for the detection of the huntingtin protein (HTT), the causative agent in Huntington's disease (HD).

HD is an autosomal dominant neurodegenerative movement and mood disorder caused by an expansion of a CAG trinucleotide repeat, to greater than 35 repeats, in exon-1 of the huntingtin gene [1]. The gene product is a ubiquitously-expressed 350 kDa HTT protein, with the greatest expression found in the central nervous system [2]. The mutant polyglutamine expanded form of HTT is cytotoxic leading to the hallmark pathology of HD, pronounced atrophy of the striatum as well as other brain regions [3].

Currently there are no disease-modifying therapies for HD, however, multiple investigators have recently reported efforts to develop therapeutic approaches that suppress huntingtin expression through RNA interference [4]–[10]. These approaches decrease the amount of mutant HTT in transgenic animals which has resulted in amelioration of HD phenotypes [11]. To enable such therapeutic programs it is crucial that the HTT protein be quantified in a reliable and robust manner to determine the pharmacodynamic effects of such potential therapeutics. To date, this has been difficult due to the structural complexity of the large HTT protein which exists in several conformers and states including soluble monomers, intermediate fibrils, insoluble aggregates, as well as several possible cleavage products [12], [13].

Here, we have developed a panel of detection assays for soluble polyglutamine-expanded (mutant) and total (polyglutamine-independent) human HTT protein as well as the rodent HTT protein ortholog using the sensitive ELISA-based Meso Scale Discovery (MSD) electrochemiluminescence assay platform [14]. We demonstrate that we are able to quantitate different forms of the polyglutamine-expanded and non-expanded HTT soluble proteins in cellular, animal, and HD patient biosamples. The MSD platform is based on the electrochemical properties of the ruthenium cation in conjunction with carbon electrode arrays held within microtiter plate footprints. A capture antibody is first non-specifically adsorbed onto the carbon surface of the plate and after analyte capture, a detection antibody labeled with the MSD SULFO-TAG ruthenium-based reagent (the electrochemiluminescent label) will generate a signal relative to the amount of analyte, such as HTT protein, present.

The advantages of this technology include high sensitivity and selectivity due to the use of two detecting antibodies, an increased dynamic range over other methods, and the ability to multiplex these assays in a high-throughput manner [15]. Alternative technologies have recently been used to quantify mutant HTT in biosamples [16]–[19]. However, each of these methods has limitations in their ability to detect some protein states; for example, one may be limited in the distance between the donor and acceptor antibody epitopes using Time-Resolved Fluorescence Resonance Energy Transfer (TR-FRET) and thus not be able to develop assays with antibody pairs that recognize the HTT protein at a considerable distance from each other; although this attribute may allow one to infer changes in conformational state which is not possible using the MSD platform. Nevertheless, this complication, along with the need to measure multiple states or species of HTT (e.g., total, expanded, and truncated) highlights the need for assays that detect HTT with antibodies that recognize different domains and are not subject to conformational effects. The MSD assays described here are amenable to detection of HTT proteins in complex fluids and tissues, which enables the implementation of multiplex measurements from single samples.

Importantly, in order to be able to quantitate the amount of HTT protein in various biosamples, we have expressed and purified both human and mouse recombinant HTT proteins with different polyglutamine lengths and have used them as reference standards in our detection assays. This has allowed us to quantitate the amount of soluble total, polyglutamine-expanded, or mouse HTT proteins in a variety of different biosamples.

We describe here three of our HTT quantification assays specific for: 1) human polyglutamine-expanded huntingtin, 2) human pan-huntingtin (polyglutamine length independent, expanded and non-expanded proteins), and 3) rodent wildtype huntingtin (endogenous mouse and rat proteins) using our novel anti-huntingtin antibodies. We show that these novel assays are able to selectively measure the amount of soluble HTT protein in a variety of biosamples including cellular, rodent, and HD patient tissues. Furthermore, we demonstrate that using our standard reference proteins, we are able to quantitatively compare the amount of HTT protein in a variety of samples which will provide guidance for dose selection and provide an ability to monitor the pharmacodynamic effects of molecular therapies and other approaches that modulation the levels of HTT protein, an important and critical step in the development of such targeted therapies.

## Materials and Methods

### Recombinant human and mouse huntingtin proteins

cDNA corresponding to the N-terminal 573 amino acid fragment of human HTT (relative to GenBank accession CAD38447.1) was amplified by PCR from full length human *HTT* cDNA in pcDNA3.1+ containing either 23 or 73 glutamine repeats. The polyglutamine tract was generated synthetically and is of a mixed codon (CAG/CAA) structure to reduce the risk of contraction or expansion during propagation and cloning. The N-terminal fragments were cloned into pPHNFLAG in frame with the N-terminal FLAG tag for expression of constructs either 571 amino acids in length (23Q) or 621 amino acids in length (73Q). The sequences of the constructs were confirmed by DNA sequencing before transfecting into Sf9 cells. Recombinant baculoviruses were generated [42], harvested, amplified, and used to infect Sf9 cells for protein production. Cells infected with *HTT* (1–573) Q23 were harvested at 72 h post infection whereas cells infected with *HTT* (1–573) Q73 were harvested at 48 h post infection due to a decrease in expression levels of *HTT* (1–573) Q73 after 48 h. Cell pellets were lysed by freeze/thaw in 50 mM Tris-HCl pH 7.4, 500 mM NaCl, 10% Glycerol, 1% CHAPS, 1 mM EDTA and protease inhibitors (Complete, EDTA-free; Roche Diagnostics). Clarification was carried out by centrifugation (22,500 rpm, 2 h, 4°C) and the soluble lysate was incubated with anti-FLAG M2 affinity gel (Sigma) overnight at 4°C on a rotator. FLAG-tagged HTT proteins were eluted with 0.4 mg/ml FLAG peptide in 50 mM Tris pH 7.4, 500 mM NaCl, 10% glycerol, 1% CHAPS, 1 mM EDTA and loaded onto a Superdex 200 16/60 column equilibrated with 50 mM Tris-HCl pH 7.4, 500 mM NaCl, 10% glycerol, 0.1% CHAPS and 1 mM EDTA. Peak fractions were analyzed by SDS-PAGE. Monomeric protein fractions were pooled and concentrated using a 5 kDa MWCO spin concentrator (Vivascience). HTT protein concentration was determined by Bradford assay. Purified HTT protein was aliquoted and stored at −80°C.

cDNA corresponding to the N-terminal 549 amino acid fragment of mouse HTT (relative to RefSeq accession number NM_010414) was amplified by PCR from full length mouse *Htt* cDNA in pPHNFLAG containing seven glutamine repeats. The N-terminal fragment was cloned into pPHNFLAG in frame with the N-terminal FLAG tag for expression of a 549 amino acid construct (7Q). The sequence of the construct was confirmed by DNA sequencing before transfecting into Sf9 cells. Recombinant baculovirus was harvested, amplified, and used to infect Sf9 cells for protein production. Cells infected with mouse *Htt* (1–549) Q7 were harvested at 72 h post infection. Cell pellets were lysed by freeze/thaw in 50 mM Tris-HCl pH 7.4, 500 mM NaCl, 10% Glycerol, 1% CHAPS, 1 mM EDTA and protease inhibitors (Complete, EDTA-free; Roche Diagnostics). Clarification was carried out by centrifugation (22,500 rpm, 2 h, 4°C) and the soluble lysate was incubated with anti-FLAG M2 affinity gel (Sigma) overnight at 4°C on a rotator. FLAG-tagged mouse HTT protein was eluted with 0.4 mg/ml FLAG peptide in 50 mM Tris-HCl pH 7.4, 150 mM NaCl and loaded onto a Superdex™ 200 16/60 column equilibrated with 50 mM Tris-HCl pH 7.4, 500 mM NaCl, 10% glycerol, 0.1% CHAPS and 1 mM EDTA. Peak fractions were analyzed by SDS-PAGE and monomeric fractions were pooled. Since the purity of the mouse HTT protein was not improved following size exclusion chromatography an ion exchange step was performed. The protein was loaded onto a MonoQ 5/50 column in a buffer containing 50 mM Tris pH 7.4, 5% glycerol, 0.1% CHAPS, 1 mM EDTA and eluted with a gradient to 500 mM NaCl over 30 column volumes. The mouse HTT protein eluted in two distinct peaks; each peak fraction was separately pooled and concentrated using a 5 kDa MWCO spin concentrator (Vivascience). Mouse HTT protein concentration was determined by Bradford assay. Purified mouse HTT protein was aliquoted and stored at −80°C.

### Anti-HTT Antibodies

Rabbit polyclonal antibodies were generated and purified using epitope peptide exclusion chromatography by the CHDI Foundation at 21^st^ Century Biochemical (Waltham, MA). Please note that, in this paper, the antibody epitope mapping is relative to GenBank accession CAD38447.1 (HTT protein carrying 25 glutamines). CHDI-90000137 (pAb137) was generated using an antigenic peptide corresponding to the HTT amino acids 4–19 (acetyl-LEKLMKAFESLKSFQQC-amide), CHDI-90000145 (pAb145) was generated using an antigenic peptide corresponding to the HTT amino acids 32–53 (acetyl-QQQQQQQQQQQPPPPPPPPPPP-Ahx-C-amide), CHDI-90000146 (pAb146) was generated using an antigenic peptide corresponding to the HTT amino acids 54–70 (human proline-rich region; acetyl-QLPQPPPQAQPLLPQPQC-amide), CHDI-90000147 (pAb147) was generated using an antigenic peptide corresponding to the HTT amino acids 37–53 of the mouse HTT (mouse proline-rich region; acetyl-pppQPPQPPPQGQPPPPC-amide) carrying seven CAG repeats (GenBank accession NP_034544), and CHDI-90000148 (pAb148) was generated using an antigenic peptide corresponding to the HTT amino acids 79–92 (acetyl-CPPGPAVAEEPLHRP-amide). The mouse monoclonal MW1 antibody against the expanded polyglutamine domain [20] was obtained from the Developmental Studies Hybridoma Bank. The mouse monoclonal MW8 antibody binding amino acid 83–90 (AEEPLHRP) near the C-terminus of exon-1 [20] was provided by the CHDI Foundation. The rabbit polyclonal BML-PW0595 antibody against amino acids 2–17 of the HTT protein was obtained from Enzo Life Science. The mouse monoclonal MAB2166 antibody, generated using a HTT fragment from amino acids 183 to 812 as a fusion protein, was obtained from Millipore. The epitope of the MAB2166 antibody has been then further mapped to a 15-amino acid region spanning from amino acid 445 to 459 of the human HTT protein [30]. A detailed overview of the antibody epitopes is reported in Table S1 in Text S1. The SULFO TAG labelling of the pAb137 antibody was performed using the MSD SULFO-TAG NHS-Ester reagent (Meso Scale Discovery, Gaithersburg, MD, USA) according to manufacturers' instructions. Preservatives in the pAb137 antibody storage buffer (e.g., sodium azide, EDTA) were removed prior to the conjugation reaction using the Zeba Spin desalting columns (Thermo Scientific) according to manufacturers' instructions. A challenge ratio of 1∶7.5 (antibody∶SULFO-TAG NHS-Ester reagent) was used for the labeling of the pAb137 antibody. The SULFO TAG-labeled pAb137 antibody was stored at −80°C in 30% glycerol.

### HD patient-derived material

HD patient-derived lymphoblast cell line carrying an HTT 73Q HD mutant allele (GM-04282) and one age-, passage number- and sex-matched control lymphoblast cell line (GM-03354) were obtained from Coriell Institute for Medical Research (New Jersey, USA). Cell culturing was performed according to protocols provided by the Coriell Institute for Medical Research. The CAG length of each cell line was verified by Laragen, Inc. [21]. For analysis in the MSD assays, approximately 2×10^7^ lymphoblast cells were lysed in 150 µl of MSD assay buffer 2 (50 mM Tris (pH 7.4), 120 mM NaCl, 1 mM EDTA, 1 mM DTT, 0.5% NP-40, 1 mM PMSF, protease inhibitors (Complete, EDTA-free; Roche Diagnostics) on ice for 15 min. Lysates were then cleared by centrifugation at 10,000× g for 15 min at 4°C and supernatants stored at −80°C until required. Total protein concentration was determined using a bicinchoninic acid assay (BCA, Thermo Scientific) according to standard procedure.

Human post mortem brain tissues including HD samples: HD-1 (Q46/17; cat# T-1991), HD-2 (Q43/19; cat# T-2019), HD-3 (Q43/17; cat# T-2476), HD-4 (Q45/16; cat# T-2959), and non-HD control samples: C-1 (cat# T-343), C-2 (cat# T-638), C-3 (cat# T-4518), were obtained from the New York Brain Bank at Columbia University (New York, USA).

### Huntington's disease *in vivo* models

Fresh snap-frozen rodent brain tissues were obtained from several sources and stored at −80°C until homogenization. If not otherwise indicated, R6/2 [22] were supplied by Dr. G. Bates (KCL, United Kingdom). Hemizygous R6/2 mice were bred in Dr. G. Bates' laboratory by backcrossing R6/2 males to (CBA×C57Bl/6) F1 (CBF) females (B6CBAF1/OlaHsd, Harlan Olac) [18]. Alternatively, R6/2 B6CBA-Tg(HDexon1)62 Gpb/1 J snap frozen brain tissues were provided by the CHDI Foundation from their colony at The Jackson Laboratories (Bar Harbor, USA). BAC HD mice [23] were obtained from the CHDI Foundation colony at the University Medical Center Hamburg-Eppendorf (UKE) and were supplied by Evotec AG (Hamburg, Germany); zQ175 C57B/L6J knock-in mice, derived from a spontaneous expansion of the CAG copy number in the CAG 140 knock-in mice [24]–[26] were provided by Evotec AG (Hamburg, Germany). Wistar (RccHan∶WIST, cat# 168) rat brain tissues were obtained from Harlan Laboratories (Horst, The Netherlands).

### Immunoblot analysis of huntingtin proteins

For immunoblotting, 25 µg of brain homogenate or 5 ng of HTT recombinant protein were mixed with 4× Laemmli loading buffer, denatured at 100°C for 5 min and loaded on the 3–8% pre-cast Tris-Acetate gels (Bio-Rad). The electrophoresis was carried out at 30 mA for 5 h (full length HTT proteins) or for 1.5 h (exon-1 HTT proteins). During long run electrophoresis, the XT Tricine Running buffer (Bio-Rad) was replaced every 1–2 h to restore the ionic content and pH. After electrophoresis, proteins were transferred to nitrocellulose membrane (Hybond-C Extra; Amersham Biosciences) in 192 mM glycine, 25 mM Tris-HCl, 20% v/v methanol for approximately 3 h at 4°C. Membranes were blocked for 1 h in PBS-T (0.1% Tween-20 in PBS) containing 5% non-fat dried milk at room temperature, washed with PBS-T, and incubated for overnight at 4°C with primary anti-HTT antibodies (in PBS-T containing 5% non-fat dried milk). Blots were washed with PBS-T, probed with HRP-linked secondary antibodies (in PBS-T with 5% non-fat dried milk) for 1 h and washed again with PBS-T. Bound antibodies were visualized using the ChemiDoc XRS Molecular Imager (Bio-Rad) according to manufacturers' instructions. Primary antibodies and dilutions were: BML-PW0595 (Enzo Life Science) (1∶3,000), pAb137, pAb147, and pAb146 (CHDI Foundation) (1∶300), pAb145 (CHDI Foundation) (1∶200), pAb148 (CHDI Foundation) (1∶1,500), MW1 (Developmental Studies Hybridoma Bank, University of Iowa) (1∶500), MAB2166 (Millipore) (1∶10,000, if not otherwise stated), MW8 (CHDI Foundation) (1∶5000), ATP5B (LSBio) (1∶20,000). HRP-conjugated secondary antibodies were as follows: goat anti-rabbit (Millipore 1∶2,000) and goat anti-mouse (Millipore 1∶500).

### MSD electrochemiluminescence assays

Dissected or whole brain human and rodent homogenates were generated using the FastPrep system (MP Biomedicals) by lysing the tissue 3×30 s in Lysing matrix tubes (Lysing matrix D; MP Biomedicals) in MSD assay buffer 1 (Tris lysis buffer (Meso Scale Discovery), Phosphatase inhibitor II 100× stock (Sigma), Phosphatase inhibitor III 100× stock (Sigma), PMSF 2 mM, protease inhibitors (Complete, EDTA-free; Roche Diagnostics), 10 mM NaF). Typically, approximately 100–150 mg of human frontal cortex and approximately 50 mg of rodent brain tissues were homogenized. Lysates were centrifuged for 20 min at 20,000× g at 4°C. Supernatant was collected, aliquoted, quickly frozen in dry ice, stored at −80°C, and used within 3–4 days to limit any detrimental effects of storage time on these samples (such as *in vitro* aggregation of HTT protein). Total protein concentration was determined using a bicinchoninic acid assay (BCA, Thermo Scientific) according to standard procedure. Besides the typical technical optimization required to develop a sensitive and robust ELISA-based assay (e.g., optimization of the antibody concentrations), the optimization of the following aspects was crucial in the development of the MSD electrochemiluminescence HTT detection assays: 1) anti-HTT antibody orientation (i.e., selection of capture and detection antibodies); 2) selection of the carbonate-bicarbonate buffer (15 mM Na_2_CO_3_/35 mM NaHCO_3_, pH 9.6) as coating buffer; 3) establish the detergent concentration compatibility to the MSD assay (e.g., Tween-20 and NP-40 are both compatible to the MSD platform up to a concentration of 0.2% and 0.5%, respectively, however other detergents are not). MSD 96-well or 384-well plates (Meso Scale Discovery) were coated overnight at 4°C with the pAb146 polyclonal antibody (expanded polyglutamine and pan human HTT assays) at a concentration of 4 µg/ml or the pAb147 polyclonal antibody (mouse HTT-specific assay) at a concentration of 8 µg/ml in carbonate-bicarbonate coating buffer (15 mM Na_2_CO_3_/35 mM NaHCO_3_, pH 9.6). Plates were then washed three times with wash buffer (0.2% Tween-20 in PBS) and blocked (2% probumin/0.2% Tween-20 in PBS) for 1 h at RT with rotational shaking and then washed three times with wash buffer. Because high detergent concentrations interfered with the ELISA, brain extracts were made up to the appropriate concentration by diluting them 5× in blocking buffer. Samples were transferred to an antibody-coated MSD plate and incubated with shaking for 1 h at RT. After removal of the lysates, the plate was washed three times with wash buffer and, depending on the assay, 25 µl (or 10 µl for 384-well plate format) MW1 (expanded polyglutamine human HTT assay) secondary antibody (diluted to 1.5 µg/ml in blocking buffer), pAb137-MSD SULFO TAG (exon-1 - pan human HTT assay), MAB2166 (pan human HTT assay) secondary antibody (diluted to 4.5 µg/ml or to 1∶7,500 in blocking buffer, respectively), or MAB2166 (mouse HTT-specific assay) secondary antibody (1∶7,500 in blocking buffer) was added to each well and incubated with shaking for 1 h at RT. In the expanded polyglutamine and in the pan human HTT assays and in the mouse HTT-specific assay, after three washes with wash buffer, 25 µl (or 10 µl for 384-well plate format) goat anti-mouse SULFO TAG detection antibody (Meso Scale Discovery) (1: 1,000 in blocking buffer) was added to each well and incubated with shaking for 1 h at RT. After washing three times with wash buffer, 150 µl (or 35 µl for 384-well plate format) of read buffer T with surfactant (Meso Scale Discovery) was added to each empty well and the plate was imaged on the SI 6000 imager (Meso Scale Discovery) according to manufacturers' instructions for 96- or 384-well plates.

Where it was necessary to use different lots of an antibody, the antibody concentration was re-optimized to generate a similar signal to background on recombinant HTT proteins as found previously (data not shown).

Total neuronal Tau protein levels were measured using a commercially available MSD ELISA-based assay kit (#K151DSA-3 Meso Scale Discovery, Gaithersburg, MD, USA) according to manufacturers' instructions.

### Competition antibody binding assay

MSD-based competition binding assays were performed in 384-well format. Wells of a bare MSD Multi-Array 384-well plate (Meso Scale Discovery) were coated with 10 µl of 4 µg/ml pAb146 or pAb147 antibody in carbonate-bicarbonate coating buffer (15 mM Na_2_CO_3_/35 mM NaHCO_3_, pH 9.6) overnight at 4°C. After washing with wash buffer (0.2% Tween-20 in PBS), wells were blocked with 35 µl of blocking buffer (2% probumin (Millipore)/0.2% Tween-20 in PBS) for 1 h at RT under shaking and washed again. Competition for binding was tested by mixing 0.1 fmol/µl human HTT (1–573) Q23 (for pAb146) or 0.1 fmol/µl mouse HTT (1–549) Q7 (for pAb147) with a concentration range from 0.001 ng/ml up to 10 µg/ml of the peptides CHDI-90000208 (acetyl-QLPQPPPQAQPLLPQPQC-amide; synthetic peptide corresponding to the proline-rich region of human HTT; epitope for pAb146 antibody), CHDI-9000209 (acetyl-PPPQPPQPPPQGQPPPPC-amide; synthetic peptide corresponding to the proline-rich region of mouse HTT; divergent from human protein sequence), and CHDI-90000210 (acetyl-CPPGPAVAEEPLHRP-amide; synthetic peptide corresponding to the C-terminus of the exon-1 region of HTT) in a mixture of 20% MSD assay buffer 2 (50 mM Tris pH 7.4, 120 mM NaCl, 1 mM EDTA, 1 mM DTT, 0.5% NP-40, 1 mM PMSF, protease inhibitors (Complete, EDTA-free; Roche Diagnostics) and 80% blocking buffer, and adding 10 µl per sample to the wells. Control wells without peptides or buffer only were included. After incubation for 1 h at RT under shaking, plates were washed and subsequently treated with 10 µl of the antibody MAB2166 (1∶1,000; Millipore), followed by 10 µl of a SULFO-tagged anti mouse antibody (1∶1,000; Meso Scale Discovery). Both antibodies were diluted in blocking buffer and in each case incubated in the wells for 1 h at RT under shaking followed by washing. After complete removal of buffer from the last wash step, 35 µl of Read Buffer T with surfactant (Meso Scale Discovery) were added to each well and plates were analyzed on a SI6000 Reader (Meso Scale Discovery) using the recommended settings for 384-well plates. Assays were performed in triplicate.

### Recombinant AAV particles for neuronal transduction

Plasmids for expression of shRNAs were modified from the AAV vector pAAV-6P-SEWB [27]. For expression of shRNAs, transcriptional control units containing the H1 promoter followed by the DNA-encoded short hairpin constructs were inserted as XbaI-XhoI fragments into pAAV-6P-SEWB to build AAV vectors with bicistronic expression units. shRNA sequences: non-allele specific *HTT* targeting shRNA sh4 (AGCTTGTCCAGGTTTATGAA; [28] and scr6 (GGCTACCTTCGTGAGTGAT), an shRNA without known target sequence in the mouse. Primary neurons were isolated from the cortex of heterozygous zQ175 [25], [26] mouse embryos (E16). The genotype of each embryo was individually determined by PCR (amplicon within transgenic neomycin cassette) and zQ175 positive cells were pooled. Typically, 2×10^5^ cells per well were seeded in 1 ml of plating medium (Minimum Essential Medium (MEM; Gibco) supplemented with 10% fetal bovine serum (Sigma-Aldrich), 2 mM L-alanyl-L-glutamine dipeptide (GlutaMAX; Life Technologies), 100 U/ml Penicillin and 100 µg/ml Streptomycin (Gibco)) into poly-L-lysine coated 24-well plates and incubated at 37°C and 5% CO_2_. 3 h after plating the medium was changed for 1 ml of culture medium (Neurobasal Medium (Gibco), 1×B27-Supplement (Invitrogen), 2 mM L-glutamine (PAA), 100 U/ml Penicillin and 100 µg/ml Streptomycin (Gibco)). Cells were cultivated for three days prior to infection with 1×10^8^ virus particles of AAV-SEWB-scr6 or AAV-SEWB-mhtt-sh4. The multiplicity of infection (MOI) based on the physical AAV titer (viral genome copies) was 500, leading to more than 90% transduced neurons which was monitored by a GFP reporter. Cells were subsequently harvested, lysed in 100 µl MSD assay buffer 2 per well (50 mM Tris pH 7.4, 120 mM NaCl, 1 mM EDTA, 1 mM DTT, 0.5% NP-40, 1 mM PMSF, protease inhibitors (Complete, EDTA-free; Roche Diagnostics), snap-frozen in liquid nitrogen, and stored at −80°C until further use.

### Statistical analysis

Unless otherwise indicated, the data in the graphs represent averages with standard deviations of the mean. Significance was calculated using a two-tailed Student's *t*-test.

### Statistical power analysis

The significance level (α) was set at 5% and the statistical power (π) at 90%. The effect of interest (difference in mean of control and test group, μ0–μ1) was set at 25% or 50%. When the variance between the control and test groups was not significantly different (F-test), the pooled variance (σ0) (mean of the %CV of the control group and the %CV of the test group) was set based on experimental observations. The size (N) of each group (assuming equal numbers of treated and control subjects) with one-sided testing was given by the formula: N = (2*[(zα−zπ)/(μ1−μ0/σ0)∧ 2])+((zα)∧ 2/4). However, when the variance between the control and test group was significantly different (F-test), the group size was calculated based on the control group variance only using the following formula: N = ([1+((100+(μ1−μ0))/100)∧ 2] * [(zα−zπ)/(μ1−μ0/σ0)∧ 2])+((zα)∧ 2/4).

The values of z come from the standardized normal distribution, depending on the chosen significance level and power. For the analysis reported in this study, with α = 0.05 and π = 0.9, zα is 1.645 and zπ is −1.282.

## Results

### Anti-huntingtin antibody characterization

**Figure 1:**
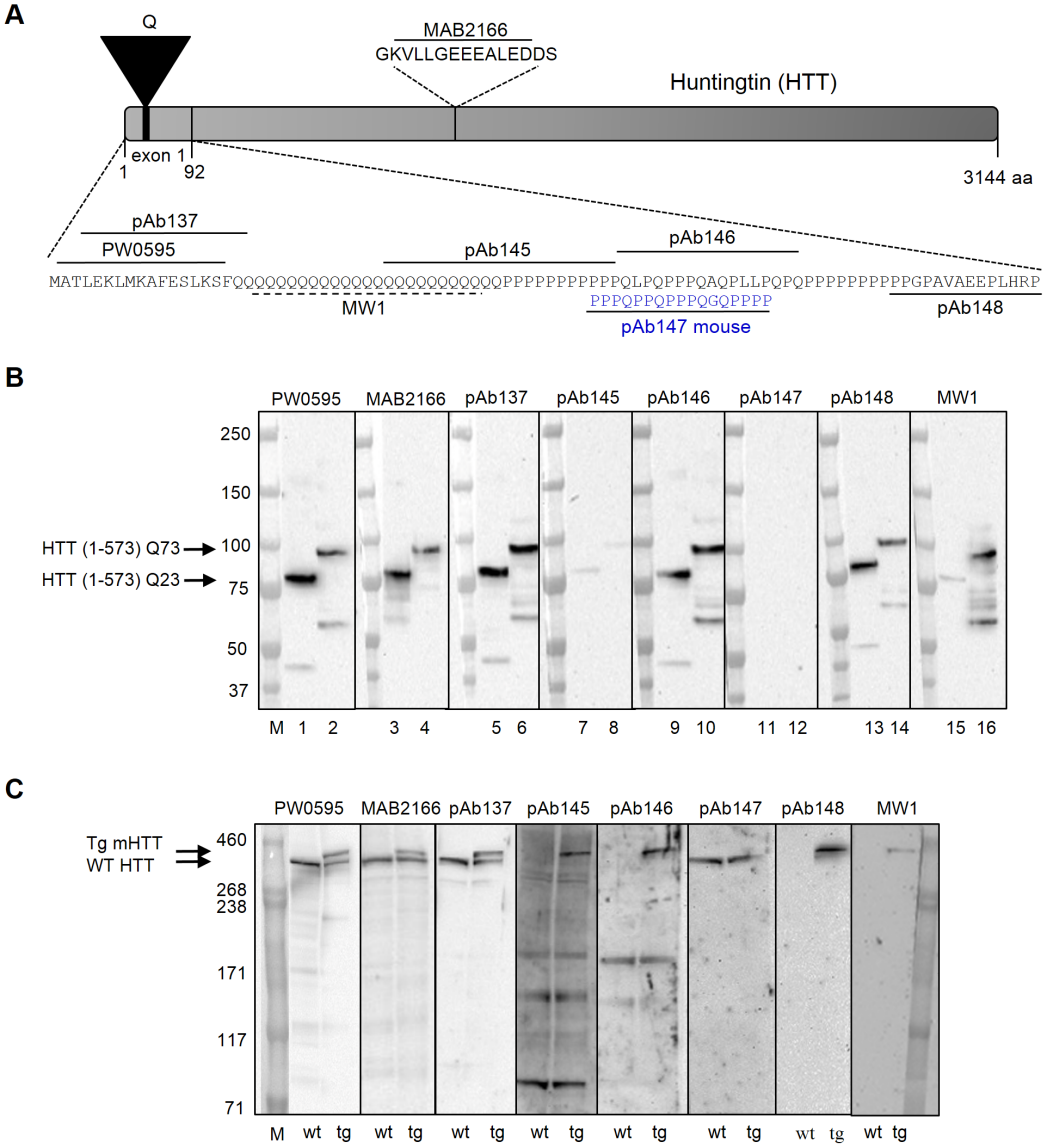
Anti-HTT antibody epitopes and analysis by immunoblot. (A) Diagram representing antibody epitopes on human HTT protein (relative to GenBank accession CAD38447.1). A stretch of glutamine (Q) residues near the N-terminus is expanded in individuals affected by Huntington's disease. Amino-acid 1-92 encoded by exon-1 are shown. Blue, pAb147 antibody epitope on mouse HTT protein (GenBank accession NP_034544). (B) Immunoblot analysis of human large fragment recombinant HTT proteins detected by the indicated anti-huntingtin antibodies. 5 ng of both HTT (1–573) Q23 (lanes 1, 3, 5, 7, 9, 11, 13 and 15) and HTT (1–573) Q73 (lanes 2, 4, 6, 8, 10, 12, 14, and 16) purified large fragment proteins were analyzed by SDS-PAGE. M, molecular weight marker (kDa). (C) Immunoblot analysis of wild type littermate and transgenic BAC HD mouse whole brain extracts. Mouse endogenous wild type and human transgenic polyglutamine-expanded HTT proteins were detected using the indicated anti-huntingtin antibodies. 25 µg of normal (wt) and transgenic (tg) mouse brain extracts were analyzed by SDS-PAGE as indicated. M, molecular weight marker (kDa). Tg mHTT, transgenic human polyglutamine-expanded HTT. WT HTT, endogenous mouse wild type HTT.

We characterized a panel of anti-HTT antibodies raised to different epitopes of the HTT protein (Figure 1A and Table S1 in Text S1) by immunoblot using purified large fragment (HTT 1–573) recombinant HTT proteins with different CAG repeat lengths (Figure 1B) as well as brain homogenates obtained from 3 month-old BAC HD mice or wild type littermates (Figure 1C). BAC HD mice express full-length human mutant HTT with 97 glutamine repeats under the control of the endogenous *Htt* regulatory machinery [23]. This animal model recapitulates several key phenotypic features of HD (e.g. late onset, atrophy of the cortex and striatum).

Each of the antibodies tested, recognized proteins at the appropriate molecular weights in gels containing both purified large fragment recombinant HTT proteins, indicated as HTT (1–573) Q23 and HTT (1–573) Q73 (Figure 1B), and BAC HD mouse brain homogenates containing the endogenous mouse HTT protein, indicated as WT HTT, and the human mutant transgene, indicated as Tg mHTT (Figure 1C). As shown in Figure 1B, the BML-PW0595, the MAB2166 [30], the pAb137, pAb145, pAb146, pAb148, and the MW1 [20] antibodies recognized both HTT (1–573) Q23 and HTT (1–573) Q73 purified large fragment proteins. The MW1 antibody, which specifically recognizes the polyglutamine domain [20], showed a higher specificity for the HTT (1–573) Q73 compared to the HTT (1–573) Q23 purified large fragment protein. The pAb147 antibody was raised against an epitope (proline-rich region) derived from the HTT mouse protein sequence that is divergent from the human HTT protein sequence and, therefore, it did not detect the human recombinant large fragment proteins. Because multiple proteins contain polyglutamine domains it was important to determine whether these antibodies bind other proteins in addition to HTT. Therefore, immunoblots of normal and transgenic mouse brain extracts were performed (Figure 1C). The antibodies tested displayed a very specific binding pattern, recognizing the HTT protein that is approximately 350 kD in size. The BML-PW0595, the MAB2166, and the pAb137 antibodies were able to detect both the lower molecular weight wild type and the higher molecular weight polyglutamine-expanded forms of HTT, whereas the pAb145, pAb146, pAb148, and the MW1 antibodies only recognized the expanded polyglutamine form present in transgenic BAC HD samples. The mouse HTT-specific pAb147 antibody only recognized, as expected, the lower molecular weight non-expanded, mouse wild type HTT endogenously expressed by both transgenic BAC HD mice and age-matched wild type littermates.

### Electrochemiluminescence assays for human huntingtin protein detection

**Figure 2:**
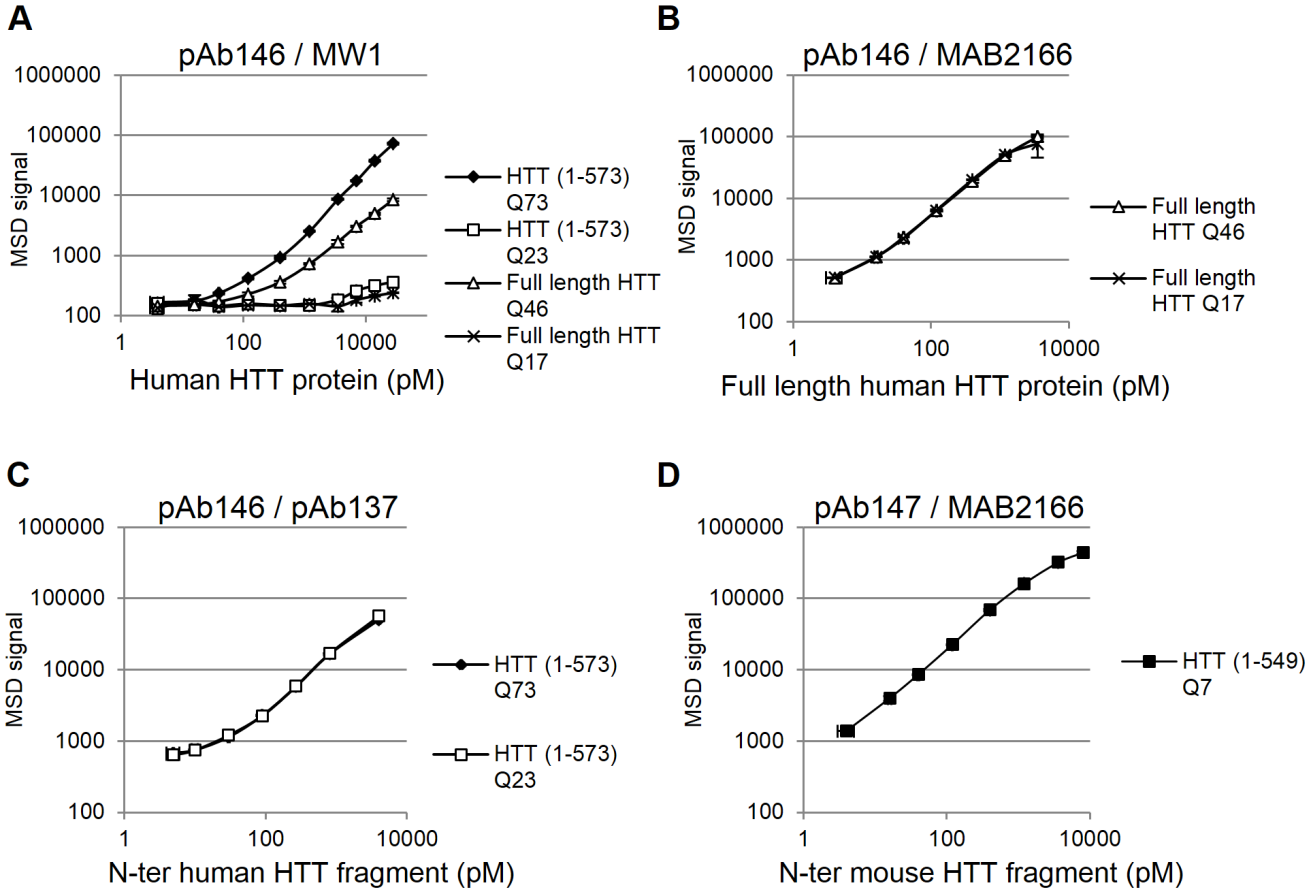
MSD assay performance with human and mouse HTT purified proteins. HTT (1–573) Q23 and HTT (1–573) Q73 large fragment and full length wild type (Q17) and mutant (Q46) recombinant human HTT proteins were spiked in the MSD assay buffer 1 at different concentrations and tested in the expanded polyglutamine human HTT MSD assay (antibody pair pAb146/MW1) (A), in the pan (antibody pair pAb146/MAB2166) (B), and in the exon-1 - pan (antibody pair pAb146/pAb137) (C) human HTT MSD assays. The HTT (1–549) Q7 mouse large fragment recombinant HTT protein was spiked in the MSD assay buffer 1 at different concentrations and tested in the mouse/rat HTT MSD assay (antibody pair pAb147/MAB2166) (D). Data are averages of n = 2 technical replicates with correspondent standard deviations.

We utilized the MSD electrochemiluminescence platform to develop a detection assay for expanded polyglutamine human HTT and an assay for pan (wild type and mutant) human HTT proteins. The pAb146 antibody was used as capture antibody in both the expanded polyglutamine human HTT and the pan human HTT assays. This antibody binds to an epitope within the proline-rich region of the human HTT protein and ensures the detection of both N-terminal fragments and full length HTT. When used in combination with MW1, a polyglutamine binding antibody, the pAb146-MW1 antibody pair specifically detected HTT (1–573) Q23 and HTT (1–573) Q73 large fragment as well as full length wild type (17Q) and mutant (46Q) [29] recombinant proteins (Figure 2A). In this assay, we used a commercially available anti-mouse MSD SULFO-TAG antibody as labeled secondary detection reagent to generate the electrochemiluminescence signal in the assay. As shown in Figure 2A, the intensity of the signal was not only dependent on the protein concentration, but also on the polyglutamine length of the proteins tested. This was expected due to the number of theoretical epitopes for the MW1 detection antibody in an expanded polyglutamine stretch [32]. Therefore, an increase in the polyglutamine length can allow for either more MW1 molecules to bind to the protein or a cooperative binding of multiple antibodies when binding sites are present in close proximity [20]. We calculated an approximately 120-fold increase in the signal for HTT (1–573) Q73 compared with HTT (1–573) Q23 measured as the ratio between the slopes calculated from the linear portion of the two standard curves (Figure 2A). The observed signal increase between full length mutant (Q46) and wild type (Q17) was approximately 23-fold. With the same calculation, we estimated approximately an 8-fold signal increase when HTT (1–573) Q73 was compared with the full length mutant (Q46) protein. The limit of detection for the MSD assay performed with the pAb146 and MW1 antibodies was calculated to be between the low nM and high pM range depending on the protein tested (Table 1).

**Table 1:** HTT MSD assay sensitivity and dynamic range.

HTT MSD assay	HTT purified protein	MSD assay lower detection limit	MSD assay dynamic range (log units)
Expanded polyQ human HTT (antibody pair: pAb146/MW1)	HTT (1–573) Q73	40 pM	2
	HTT (1–573) Q23	8 nM	N/A
	FL HTT Q46	120 pM	1.5
	FL HTT Q17	16 nM	N/A
Pan human HTT (antibody pair: pAb146/MAB2166)	FL HTT Q46	<4 pM	2
	FL HTT Q17	<4 pM	2
Exon-1 – pan human HTT (antibody pair: pAb146/pAb137)	HTT (1–573) Q73	∼4 pM	1.5
	HTT (1–573) Q23	∼4 pM	1.5
Mouse/Rat HTT (antibody pair: pAb147/MAB2166)	HTT (1–549) Q7	<4 pM	2.5

*Note*. Lower detection limit was determined from the calibration curve using the calculation of background mean +3× Standard deviation. Log units were defined by visual interpretation.

Antibody pairs are indicated as capture/detection antibody.

All together, these results demonstrated a specific detection of human HTT protein in a polyglutamine-dependent manner by using the antibody pair pAb146-MW1 in an electrochemiluminescence assay. Both large N-terminal fragments and full length polyglutamine-expanded HTT proteins were detected with a high sensitivity and specificity and across a broad dynamic range of concentrations.

**Figure 3:**
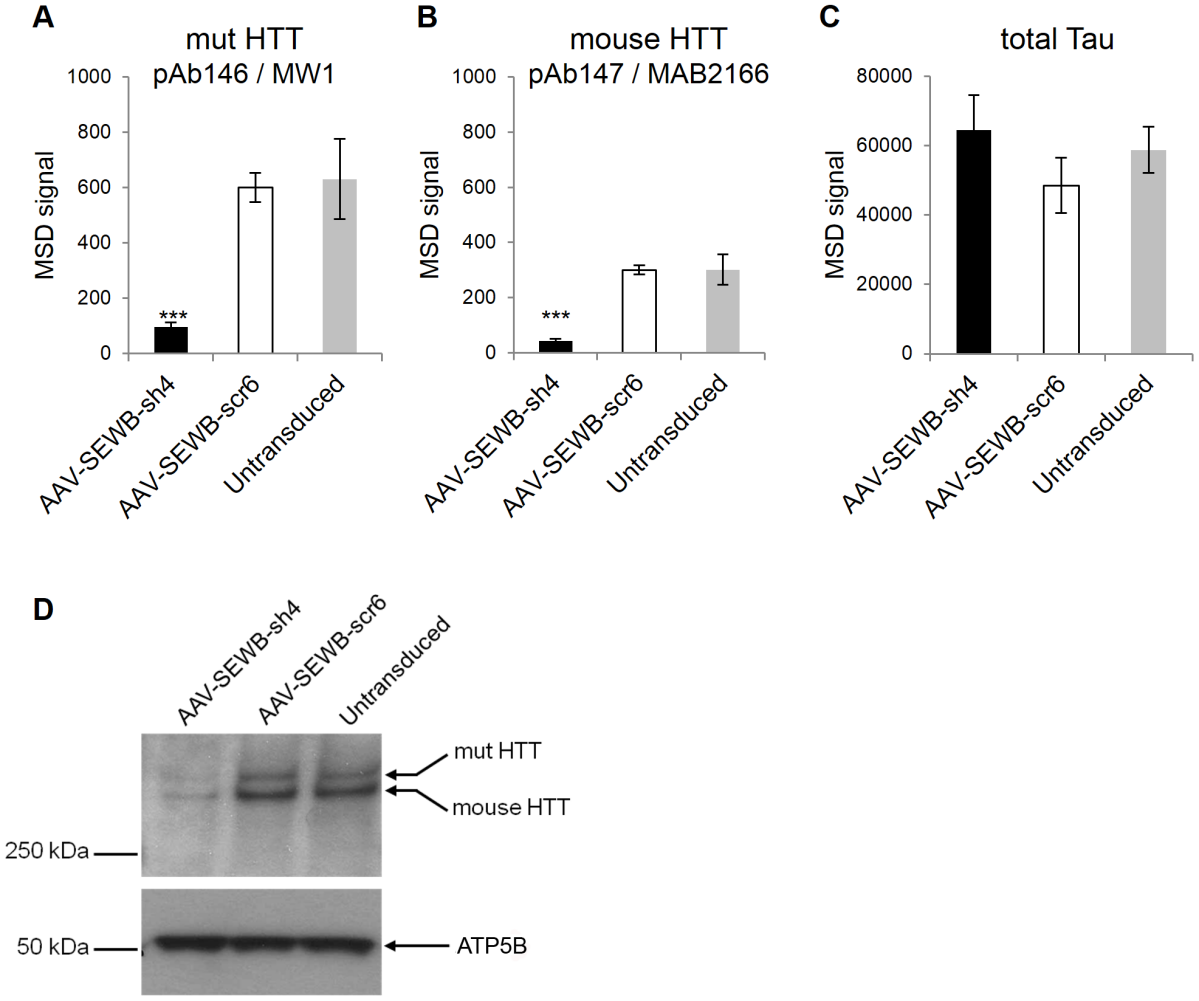
Specificity of human mutant and mouse HTT detection. The adeno-associated AAV-shRNA expression vector AAV-SEWB-sh4 was transduced into heterozygous zQ175 mouse primary neurons and humanized mutant (A) or endogenous mouse (B) HTT proteins were evaluated using the expanded polyglutamine human HTT MSD assay (antibody pair pAb146/MW1) or the mouse/rat HTT MSD assay (antibody pair pAb147/MAB2166), respectively. sh4, *HTT* targeting shRNA. scr6, scramble control shRNA. (C) Neuronal total tau protein levels measured using a commercially available MSD ELISA-based assay kit were monitored as loading control. Data are averages of n = 3 independent samples with correspondent standard deviations. ***, P<0.001. (D) Immunoblot confirming the AAV-mediated knockdown of humanized mutant (mut) and endogenous mouse HTT in transduced heterozygous zQ175 mouse primary neurons (AAV-SEWB-sh4: *HTT* targeting shRNA; AAV-SEWB-scr6: scrambled control shRNA). Immunoblot was probed for HTT (MAB2166, 1∶1,000; Millipore) or ATP5B as loading control.

The specificity of the human mutant HTT signal detection by the pAb146-MW1 MSD assay was further confirmed in heterozygous zQ175 knock-in mouse primary neurons after adeno-associated virus (AAV) - mediated shRNA knockdown (Figure 3A). We detected a significant signal reduction in neurons transduced with the *HTT*-specific AAV-SEWB-sh4 [28], confirming the specificity of the human mutant HTT signal detected by the expanded polyglutamine human HTT MSD assay. When quantified as percentage of signal versus the control transduction (AAV-SEWB-scr6), the mutant human HTT signal in primary neurons transduced with the *HTT*-specific AAV-SEWB-sh4 was estimated to be reduced by 84%. Detection of neuronal total tau in the same zQ175 primary neuron lysates, monitored using a commercially available MSD ELISA-based assay, was used as sample normalizer (Figure 3C). These results were also confirmed by immunoblot (Figure 3D).

The same MSD ELISA-based platform was used to develop the pan (expanded mutant and wild type), or “total” human HTT assay. For this assay, the polyclonal pAb146 antibody was again used as a capture antibody. This antibody was used in combination with the MAB2166, a mouse monoclonal antibody raised against a fusion protein encompassing the region from amino acid 183 to 812 of the HTT protein. The target epitope of this antibody has been subsequently narrowed down to a region spanning amino acid 445 to 459 of the human HTT protein [30]. The assay performed using the pAb146-MAB2166 antibody combination enables the detection of more full length wild type (Q17) and mutant (Q46) HTT proteins (Figure 2B) as defined by the distant antibody epitopes. As shown in Figure 2B, the very good overlap of the two standard curves indicated that this assay can detect human HTT proteins independently of the length of the polyglutamine tract of the proteins. This was expected because both capture and detection antibodies target epitopes located outside the polyglutamine region. The limit of detection of the assay performed using the pAb146-MAB2166 antibodies was determined to be in the low pM range for both full length HTT proteins tested (Table 1).

The target epitope of the monoclonal MAB2166 antibody maps outside the HTT protein sequence encoded by the exon-1 of the HD gene and this makes the pan, polyglutamine length-independent, human HTT assay developed with the pAb146 and MAB2166 antibodies not suited for detection of HTT exon-1 protein fragments. Therefore, we developed an alternative version of the pan human HTT assay that allows for the detection of shorter HTT fragments. The MAB2166 detection antibody was replaced by pAb137, an antibody raised to an epitope in the N-terminal region (amino acid 4-19) of the HTT protein (Figure 1A) and pAb146 remained the capture antibody. To enable the use of two rabbit antibodies in the same assay, the pAb137 detection antibody was directly labeled using the SULFO-TAG NHS-ester labeling reagent available from Meso Scale Discovery. The assay performed using the pAb146-pAb137 antibody combination enables the specific detection of HTT (1–573) Q23 and HTT (1–573) Q73 large fragment HTT proteins (Figure 2C). Also in this version of the assay, the detection of the human large fragment HTT proteins appeared to be completely independent of the length of the polyglutamine tract. The signal detected using the pAb146-pAb137 antibodies was slightly lower compared to that seen when using the pAb146-MAB2166 antibody combination. Also of note is that the assay sensitivity appeared to be slightly lower, although still in the low pM range (Table 1).

For simplicity, in this paper we refer to the MSD assay developed using the pAb146-MAB2166 antibody combination as pan (i.e., polyQ-independent) human HTT assay and the MSD assay for which the pAb146-pAb137 antibody combination as exon-1 - pan human HTT assay.

### Electrochemiluminescence assay for rodent huntingtin protein detection

The MSD electrochemiluminescence platform was also used to develop a detection assay for endogenously expressed mouse HTT protein. The mouse HTT-selective pAb147 antibody, which binds to an epitope within the proline-rich region of the mouse HTT protein, was used as capture antibody. The MAB2166 antibody was used as detection antibody. The epitope of this antibody maps to a 15-amino acid region spanning from amino acid 445 to 459, a conserved region between human and mouse HTT proteins [30]. In this assay, we used a commercially available anti-mouse MSD SULFO-TAG antibody as labeled secondary detection reagent to generate the electrochemiluminescence in the assay. The mouse HTT-specific assay functionality was confirmed using the purified mouse HTT (1–549) Q7 large fragment protein (Figure 2D) and sensitivity for this assay was estimated to be in the low pM range (Table 1).

To confirm the specificity of the mouse HTT signal detection by the pAb147-MAB2166 MSD assay, the endogenous mouse HTT protein was monitored in zQ175 mouse primary neurons after adeno-associated virus (AAV)-mediated shRNA knockdown (Figure 3B). The effect of the *HTT*-specific AAV-SEWB-sh4 [28] on the mouse HTT protein signal was clearly detected by the mouse HTT-specific ELISA-based MSD assay. When quantified as a percentage of signal versus control transduction (AAV-SEWB-scr6), the mouse HTT signal in heterozygous zQ175 primary neurons transduced with the *HTT*-specific AAV-SEWB-sh4 was found to be reduced by 86%. Detection of neuronal total tau in the zQ175 primary neuron lysates, monitored using a commercially available MSD ELISA-based assay, was used as sample normalizer (Figure 3C).

For an evaluation of potential species cross-reactivity effects of the used capture antibodies pAb146 and pAb147, competition binding assays between human HTT (1–573) Q23 or mouse HTT (1–549) Q7 large fragment proteins and various peptides were performed. The human peptide CHDI-90000208 represents the epitope for pAb146, CHDI-90000209 is the mouse equivalent of CHDI-90000208 which represents the epitope for pAb147, and CHDI-90000210 is a peptide corresponding to an unrelated region of human HTT. The competition binding assay showed that only the human peptide CHDI-90000208 competes with human HTT (1–573) Q23 protein for binding to the capture antibody pAb146, neither the mouse peptide CHDI-9000209 nor the unrelated peptide CHDI-90000210 showed any significant competitive effect (Figure S1A). Conversely, the mouse peptide CHDI-90000209 was the only peptide to compete for binding to pAb147 (Figure S1B); albeit the mouse peptide displayed a slight 10% inhibition of binding at the highest concentration tested (10 mM). These data demonstrate that the pAb146 and pAb147 antibodies are selective for the respective species with little to no cross-reactivity.

We also tested whether the MSD assay performed with the pAb147 and MAB2166 could be used to detect endogenous HTT in rat brain tissue as the target epitope of both antibodies is completely conserved between mouse and rat HTT proteins. Extracts generated from RccHan∶WIST wild type rat brains were prepared and tested in the pAb147-MAB2166 HTT MSD assay and showed a significant signal over background (Figure S2), whereas human-derived cortical tissue extracts containing human HTT used as negative control showed a signal at background level, confirming that this MSD assay can be used to monitor both mouse and rat HTT proteins, but not human HTT.

### Assessment of huntingtin levels in biological samples

Our ultimate goal is to develop assays that allow the detection and quantification of HTT proteins in biological samples. The added complexity of cell lysates and tissue homogenate extracts may influence antibody binding and affect the overall performance of the assay. In order to establish the effect of the biological sample matrix on the MSD assays, we performed a spike-and-recovery experiment. A constant amount (20 micrograms) of mouse brain homogenate generated from a (CBA×C57Bl/6) F1 (CBF) (B6CBAF1/OlaHsd, Harlan Olac) wild type mouse was spiked with increasing concentrations of recombinant human HTT (1–573) Q73 and HTT (1–573) Q23 proteins. The samples were tested in both the expanded polyglutamine and the exon-1 - pan human HTT MSD assays (Figure S3A and S3C, respectively). The effect of the biological sample matrix was also tested on the signal recovery of the purified full length human HTT Q46 and HTT Q17 proteins in the pan human HTT assay (Figure S3B). Because the developed assays are specific for human HTT we did not expect, nor did we observe, any interference by the endogenous mouse HTT protein present in the brain extract.

The same assessment was carried out for the mouse HTT-specific MSD assay but, in this case, the recombinant mouse HTT (1–549) Q7 protein was spiked in a brain homogenate obtained from a 3 month-old homozygous zQ175 mouse which carries two chimeric huntingtin alleles comprised of human exon-1 knocked into the mouse gene (Figure S3D). Because the sequence of the exon-1 polyproline domain of this chimeric HTT protein is derived from human HTT, we would not expect any detection by the mouse polyproline sequence-specific pAb147 antibody.

The performance of all samples in the different MSD assays was compared with that of recombinant HTT proteins spiked in assay buffer diluent. In order to accurately evaluate the effect of the sample biological matrix, the same lysis buffer (MSD assay buffer 1) used as diluent for the HTT recombinant proteins was also used to generate the mouse brain extracts. An alternative lysis buffer (MSD assay buffer 2) was also tested for the human HTT recombinant fragments (see Figure S4) and overall results obtained with the two different lysis buffers were found to be comparable. The influence of the sample biological matrix on all of the MSD assays was minimal. The limit of detection for all the MSD assays performed in brain lysates did not substantially differ from that calculated for the recombinant HTT proteins spiked in assay buffer and, in agreement with what was observed previously, was estimated to be in the pM range, independently from the presence of the sample biological matrix (Figure S3A–D).

### Detection and quantification of soluble HTT in brain tissues of HD *in vivo* models

To establish whether the pAb146-MW1 MSD HTT detection assay is suitable to measure mutant HTT levels in tissue samples, we generated brain tissue homogenates from different transgenic and knock-in HD mouse models and tested them in the HTT MSD assays.

**Figure 4:**
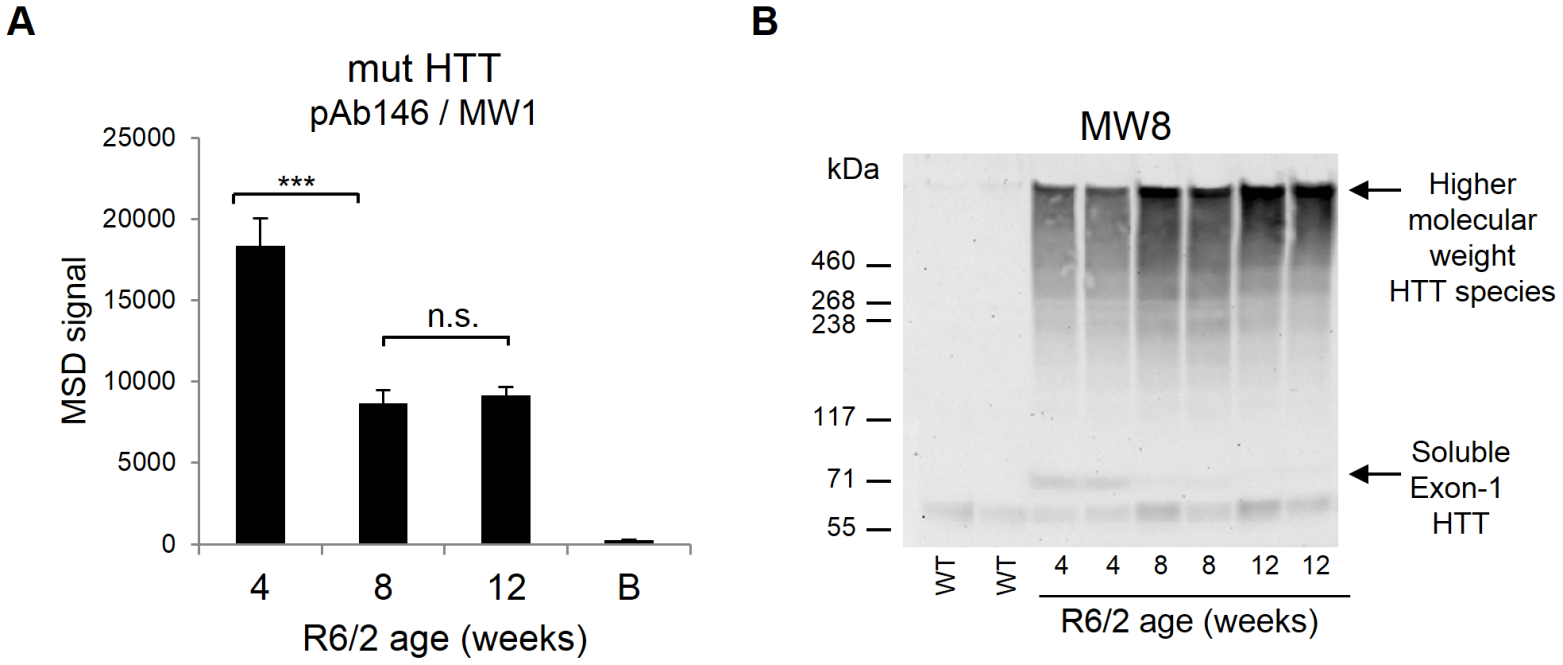
Decrease of soluble mutant HTT levels in R6/2 brain tissues is associated with increased age of the mice. (A) Homogenates from brains of R6/2 female mice obtained from The Jackson Laboratory (bearing an expanded polyglutamine tract of 120 CAG repeats on average) were analyzed for detection of soluble mutant human HTT at different ages (4, 8 and 12 weeks). Tissues analyzed showed significant signals in the expanded polyglutamine human HTT MSD assay (antibody pair pAb146/MW1) and a significant signal decrease between 4 and 8 weeks of age. Data are averages of n = 4 independent samples with correspondent standard deviations. B, assay background. ***, P<0.001. (B) SDS-PAGE and immunoblotting for HTT with the MW8 antibody reveals high molecular weight bands (presumably HTT aggregates) in R6/2 brain homogenates that increase with increasing age of the animals. Progressive decrease of soluble monomeric HTT fragments can be observed. WT, wild type (CBA×C57Bl/6) F1 (CBF) (B6CBAF1/OlaHsd, Harlan Olac) mice.

Brain tissue homogenates obtained from 4, 8 and 12 week-old R6/2 mice were analyzed using the expanded polyglutamine human HTT MSD assay. This transgenic mouse model contains a 1.9-kb fragment encompassing the human HTT promoter and exon-1 [22] bearing an expanded polyglutamine tract of 120 CAG repeats on average in the colony used. The R6/2 mice exhibit both early and severe behavioral and anatomical symptoms [19], [22]. Significant signals were observed for all transgenic R6/2 tissue samples analyzed (Figure 4A). In addition, tissue homogenates showed a decreased signal with increased age of the R6/2 mice. We measured a 2-fold signal decrease for 8 week-old brains compared with 4 week-old R6/2 tissues, whereas no additional signal reduction was seen for 12 week-old R6/2 samples. This signal decrease is most likely caused by a shift of mutant HTT exon-1 fragment protein from a soluble to aggregated form as a function of disease progression as previously reported [36] and was also observed in immunoblot analysis of these tissues (Figure 4B). Similar results were seen when micro-dissected cortex, striatum and brain stem tissue homogenates obtained from 4, 8, 12, and 15 week-old R6/2 mice of a different colony (bearing an expanded polyglutamine tract of 206 CAG repeats on average) were analyzed using the expanded polyglutamine human HTT MSD assay (Figure S5). The overall signals observed for cortex and striatum samples were comparable, whereas those detected for the brain stem samples were lower, especially for the 4 and 8 week time-points, suggesting lower levels of HTT exon-1 expression in this tissue. The most significant signal decrease was observed up to 12 weeks where we we measured a 2.7-, 3-, and 2-fold signal decrease respectively for 12 week-old cortex, striatum, and brain stem tissues compared with 4 week-old R6/2 tissues. The signals detected for 12 and 15 week time-points were comparable for all tissues tested. These data demonstrated that the pAb146-MW1 MSD HTT assay is specific for predominantly soluble forms of mutant HTT.

**Figure 5:**
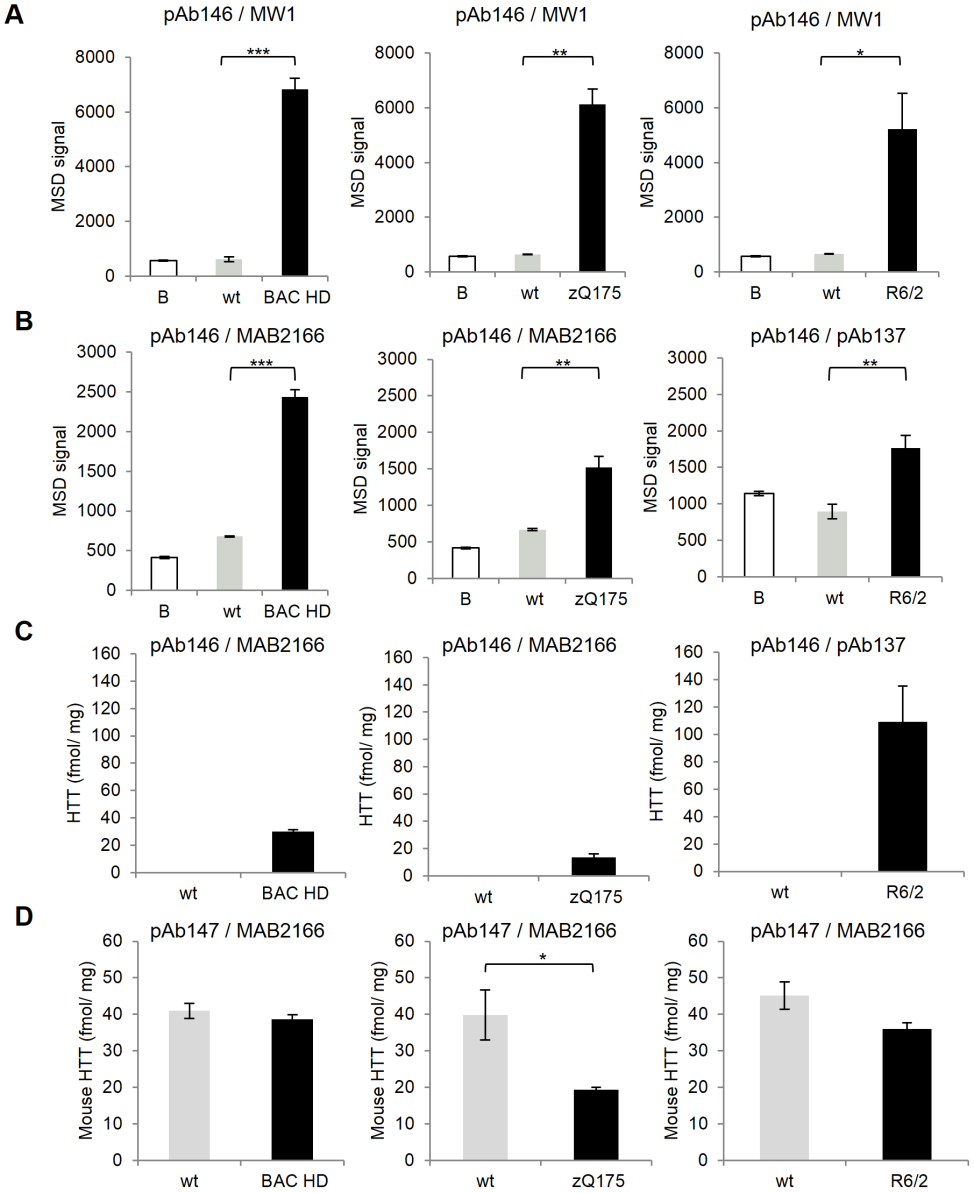
Quantification of mutant human HTT and endogenous mouse HTT detection in brain extracts derived from three different mouse HD models. Human mutant HTT proteins expressed in 3 month-old BAC HD, 4 week-old R6/2 and 3 month-old heterozygous zQ175 mouse models were detected using the expanded polyglutamine human HTT MSD assay (antibody pair pAb146/MW1) (A) and the pan human HTT MSD assay (antibody pair pAb146/MAB2166) for BAC HD and zQ175 mice or the exon-1 - pan human HTT MSD assay (antibody pair pAb146/pAb137) for R6/2 mice (B). A total of 20 µg of whole brain tissue homogenates were used for the analysis. Correspondent wild type age-matched control animals (wt) were included as negative controls. (C) Human mutant HTT proteins in the above mentioned HD mouse models were quantified using standard curves of large fragment (exon-1 - pan human HTT MSD assay) or full length HTT recombinant proteins (pan human HTT MSD assay) as standards. (D) The expression of mouse endogenous HTT was detected using the mouse/rat HTT MSD assay (antibody pair pAb147/MAB2166) and quantified using standard curves of purified HTT (1–549) large fragment mouse HTT protein. Quantified human HTT and mouse HTT proteins are expressed as femtomoles per milligrams of total brain extract input protein. Data are averages of n = 3 independent samples with correspondent standard deviations. B, assay background. *, P<0.05; **, P<0.01; ***, P<0.001.

The ability of the MSD HTT assays to detect mutant HTT levels in tissue samples was further assessed using whole brain homogenates obtained from several HD rodent models. Tissues obtained from 3 month-old BAC HD mice [23], 3 month-old heterozygous zQ175 knock-in mice [25], [26] and their correspondent wild type littermates were homogenized and assessed in the MSD assay performed using the pAb146-MW1 antibody combination for specific detection of the mutant human HTT protein (Figure 5A). Hemispheres of 4 week-old R6/2 brain extracts were also included in the analysis and levels of transgenic HTT detection compared to those in age-match wild type controls (Figure 5A). Specific detection of mutant full length human HTT proteins were detected in the BAC-HD and z_Q175 mice as well as the human HTT exon-1 fragment protein in R6/2 mice when compared to wild type controls. An approximately 10-fold higher MSD signal was seen in both BAC HD and zQ175 mouse models compared to the correspondent age-matched control mice. A slightly lower signal increase was detected in the R6/2 mouse model (approximately 8-fold higher compared to age-matched control mice). This result confirmed the utility of this assay for detection of soluble full length and fragment forms of mutant HTT in different HD mouse models.

The brain tissue extracts generated from the different HD mouse models were also analyzed in the pan human HTT MSD assay (BAC HD and zQ175 mice) or in the exon-1 - pan human HTT MSD assay (R6/2 mice) (Figure 5B). Because these models express only mutant human HTT, the results showed the same pattern as that evidenced when samples were assessed in the expanded polyglutamine human HTT assay (see also Figure 5A). However, because the pan human HTT assays detect human HTT proteins independently of polyglutamine repeat-length, the use of these assays also allows an accurate quantification of the human mutant HTT protein expression levels in the samples. On the other hand, the availability of recombinant HTT proteins carrying a significantly different polyglutamine length compared to that of HTT proteins expressed by the HD mouse models makes the estimation of HTT concentration in the expanded polyglutamine human HTT MSD assay inaccurate without using a CAG repeat length correction factor. We therefore employed the exon-1 - pan human HTT assay and used the HTT (1–573) Q73 fragment to generate standard curves to quantify human mutant exon-1 HTT expression levels in R6/2 mice. The purified full length Q46 HTT protein was used as standard reference in the pan human HTT assay for the quantification of human mutant HTT in both BAC HD and zQ175 full length mouse models. Although the effect of the sample biological matrix on the pan human HTT assay signal recovery was previously shown to be minimal (see also Figure S3), the recombinant HTT proteins were spiked in mouse brain homogenate generated from wild type age-matched control mice to account for any effects, although minimal, of the sample biological matrix. Between the two mouse models expressing full length HTT, the transgenic BAC HD model, likely to carry multiple copies of the transgene, showed higher levels of mutant human HTT protein compared to heterozygous zQ175 knock-in mice, which only carry one chimeric mouse/human exon-1 allele (Figure 5C).

We also quantified the levels of endogenous mouse HTT in the different animal models using the pAb147-MAB2166 antibody combination for specific detection of the endogenous mouse HTT protein and used the purified mouse HTT (1–549) Q7 large fragment protein as the standard (Figure 5D). Both BAC HD and R6/2 transgenic animal models showed comparable levels of endogenous mouse HTT expression, which, as expected, were also found to be very similar to those detected in wild type age-matched control mice. A significant difference (approximately 50%) in endogenous mouse HTT expression was seen between heterozygous zQ175 mice and the correspondent wild type controls. This was expected because, as previously mentioned, the heterozygous zQ175 mice carry only one complete mouse *HTT* allele.

### Detection of HTT protein in human samples

**Figure 6:**
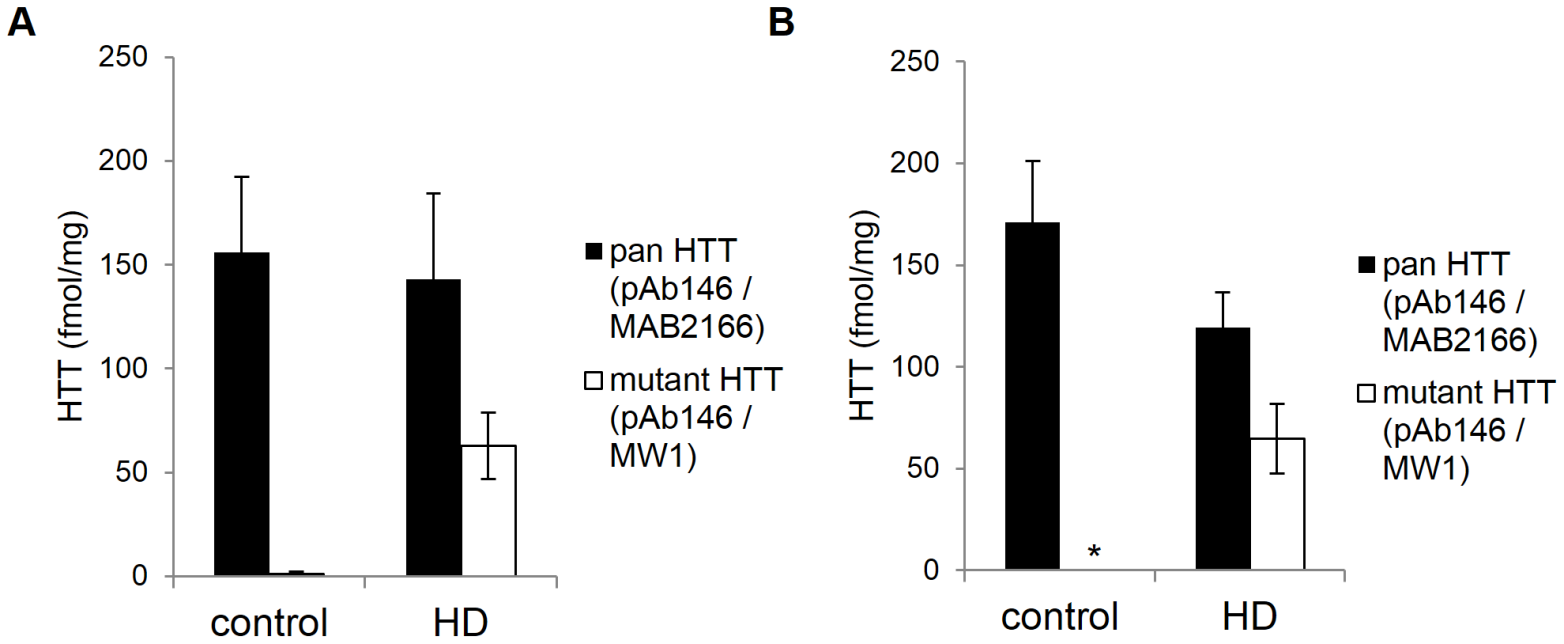
Detection of polyglutamine-expanded (antibody pair pAb146/MW1) and non-expanded (antibody pair pAb146/MAB2166) human HTT proteins in HD patient lymphoblast cell lines and human brain tissues. (A) Detection and quantification of human HTT proteins in HD and non-HD control patient lymphoblast lysates. Human HTT proteins were quantified using standard curves of HTT (1–573) Q73 recombinant protein. Technical triplicates with standard deviations are shown. 1 mg/ml of total protein lysates was tested. (B) Detection and quantification of human HTT proteins in post mortem frontal cortex homogenates of 4 HD patient and 3 non-HD control donors. Human HTT proteins were quantified using standard curves of human full length Q46 HTT recombinant protein. Data are presented as average values with correspondent standard deviations. Asterisk marks HTT levels below the limit of detection. 0.5 mg/ml of total protein homogenates was tested. For the analysis of the human brain extracts in the HTT MSD assays, the MW1 detection antibody was used at a concentration of 7.5 µg/ml and the MAB2166 detection antibody was used at a dilution of 1∶1,000.

Because the sensitivity and specificity of the human HTT MSD assays were successfully demonstrated for human *HTT* transgenes in rodent brain tissues, we next focused on the detection of polyglutamine expanded and non-expanded HTT in a clinically relevant setting and analyzed human lymphoblast cell lines and post mortem cortex tissues obtained from HD and age-matched healthy control patients. These samples offered the opportunity to also evaluate the performance of the pan human HTT assay which was not possible to investigate in the animal samples not expressing wild type human HTT. HD (GM-04282 Q73/19) and non-HD control (GM-03354 Q23/19) lymphoblast samples analyzed in the pan human HTT assay showed comparable total (polyglutamine expanded and non-expanded) levels of HTT expression; 143.1±41.2 and 155.9±36.6 femtomoles HTT per milligram total input protein, respectively (Figure 6A). Healthy control patient lymphoblasts showed, as expected, a signal close to background levels when tested in the expanded polyglutamine human HTT MSD assay, whereas mutant HTT expression level in HD patient lymphoblasts was found to be approximately 44% lower (63.0±16.0 femtomoles mutant HTT per milligram total input protein) compared to the correspondent total HTT amount. Quantification of mutant HTT levels was performed using the purified human HTT (1–573) Q73 large fragment protein as standard reference. The estimation of mutant HTT expression levels is in this case accurate, because both standard and endogenously expressed mutant HTT proteins carry the same CAG repeat length. Post mortem cortex homogenates of HD patients (HD-1 (Q46/17), HD-2 (Q43/19), HD-3 (Q43/17), HD-4 (Q45/16)) and non-HD control donors (C-1, C-2, C-3) were tested and estimated amounts of HTT for each sample were calculated using standard curves of recombinant full length human mutant (Q46) HTT protein (Figure 6B). Also in this case, the estimation of mutant HTT expression levels is accurate because both standard and endogenously expressed mutant HTT proteins carry approximately the same CAG repeat length. Amounts of total HTT vary between 100 and 190 femtomoles per milligram total input protein across all tested samples, with average values of 171.2±30.0 femtomoles HTT per milligram total input protein in non-HD control samples and 119.2±17.5 femtomoles HTT per milligram total input protein in HD samples. Polyglutamine-expanded HTT could only be detected in the HD homogenates with an average value of 64.8±17.1 femtomoles mutant HTT per milligram total input protein, which is equivalent to approximately 54% of the respective amount of total HTT.

An overview of the HD patient details and the corresponding estimated mutant and pan HTT amounts, based on data generated using the HTT MSD assays, is reported in Table 2.

**Table 2:** Human sample overview.

Sample	Age (yrs)	Gender	Diagnosis	CAG Repeats	Cold PMI	Frozen PMI	Mut HTT (fmol/mg)	Total HTT (fmol/mg)
**C-1**	62	male	No diagnostic abnormality recognized	n/a	04:16	08:16	N/A	188.8±38.3
**C-2**	78	male	No diagnostic abnormality recognized	n/a	05:20	08:00	N/A	136.6±2.3
**C-3**	56	male	Control	n/a	n/a	08:50	N/A	188.2±20.8
**HD-1**	53	male	Huntington's Disease, Vonsattel grade 3/4	46/17	06:45	11:17	77.0±18.0	125.8±5.4
**HD-2**	61	male	Huntington's Disease, Vonsattel grade 3/4	43/19	17:05	18:25	57.9±10.6	140.4±13.8
**HD-3**	60	male	Huntington's Disease, Vonsattel grade 3/4	43/17	00:00	22:55	43.9±10.0	109.8±10.0
**HD-4**	47	male	Huntington's Disease, Vonsattel grade 3/4	45/16	02:20	11:40	80.7±22.2	100.9±14.6
**GM-04282**	20	female	Huntington's Disease onset at age 14 yrs	73/19	N/A	N/A	63.0±16.0	143.1±41.2
**GM-03354**	25	female	Diabetes Mellitus, juvenile onset	23/19	N/A	N/A	N/A	155.9±36.6

*Note*. HTT amounts are intended per mg of total input protein in the homogenate.

Cold PMI: Post mortem interval calculated from the patients reported time of death to the time the patient was brought into the cold room.

Frozen PMI: Post mortem interval calculated from the patients reported time of death to the time the brain was processed.

## Discussion

We have developed a panel of versatile and highly sensitive ELISA-based bioassays utilizing the MSD electrochemiluminescence platform to quantify soluble polyglutamine-expanded and non-expanded human HTT protein in animal and human tissues. Additionally, we have developed an assay that specifically detects soluble mouse and rat HTT proteins. Importantly, we have generated both human and mouse recombinant huntingtin fragment proteins as references to estimate the quantity of human HTT and mouse HTT in biological samples. The use of the MSD technology, based on stable electrochemiluminescent labels, ensures that only labels near the electrode are excited and detected leading to precise and reproducible quantitation of HTT. In addition, the multiple excitation cycles of each label amplify the signal to enhance light levels and improve sensitivity; all assays described can detect HTT proteins in the low pM range. The assays are conducted in high-throughput 96- or 384-well plate format and, contrary to other assays using alternative technologies to measure HTT in biosamples [16], [17], [19], [36], in principle they can be multiplexed to evaluate HTT levels with respect to other unrelated proteins. The assays are also relatively resistant to changes in biological matrix (e.g., whole brain homogenate) and thereby allows for the comparison of diverse preclinical samples.

We have successfully quantified HTT proteins in different rodent HD models using our bioassays. Of the two full-length HTT-expressing mouse models analyzed, the BAC HD mice showed higher levels of mutant HTT expression compared to the heterozygous zQ175 KI mice, in line with what one might expect from a mouse model carrying multiple transgenic copies of exogenously derived mutant HTT. In R6/2 mice, the level of soluble exon-1 mutant HTT in whole brains and different brain regions was significantly lower in older symptomatic mice than in younger pre-symptomatic mice. This has been attributed to a progressive shift from the soluble to aggregated form of HTT which correlates with disease progression [31]. This highlights the point that our assays detect soluble HTT and not higher molecular species of HTT such as aggregates. Therefore, when employing these assays one must keep in mind that the amounts measured do not include those higher molecular species.

The mouse monoclonal antibody MW1, used as detection antibody in our expanded polyglutamine HTT assay, binds to expanded polyglutamine tracts in a linear lattice model [32] and its epitope is rapidly lost upon aggregation [13]. In line with these findings, we concluded that our expanded polyglutamine-HTT MSD assay specifically detects soluble forms of HTT, which are progressively recruited into non-detectable insoluble species with the progression of the disease phenotype in the R6/2 HD mouse model. To specifically detect early aggregated species of HTT, alternative assays such as the Seprion assay [18] or the MW8/MW8 TR-FRET assay [33] are highly complementary to the assays described here. Absolute quantification with these assays will, however, be very challenging since the generation of an aggregated protein standard may prove difficult as this is a Q length-, time-, and concentration-dependent process *in vitro* [34].

The use of MW1 as a detection antibody has resulted in the MSD signals of the expanded polyglutamine human HTT MSD assay directly proportional to the CAG length of the HTT proteins analyzed. Data in this paper and our unpublished data from congenic animal models with different CAG expansions between 100 and 270 repeats suggest that the signal increase with CAG expansion remains linear throughout this range. This is in contrast to the HTT TR-FRET assay, which appears to demonstrate a plateau in CAG expansion-mediated signal increase around 60 repeats [35]. Since we have utilized recombinant HTT proteins with CAG-repeat length within the range of both human wild type (17Q and 23Q) and human expanded (46Q and 73Q) alleles, we were able to estimate the HTT concentration in human biosamples using our MSD assays. However, because the majority of the HD mouse models carry a far larger polyglutamine tract than the currently available purified standard proteins, HTT quantification using the expanded-polyglutamine human HTT MSD bioassay leads to an inaccurate HTT protein estimation, even when the linear correction factor for CAG expansion is used. Therefore, we developed a polyglutamine-length-independent MSD assay (which should detect all soluble HTT conformers) in human biosamples which ensures accurate quantification of human mutant HTT protein expressed in HD mouse models. Importantly, we have optimized two different versions of this assay to quantify both full length and exon-1 human HTT proteins in respective HD models. Recent findings suggest that the HTT exon-1 fragment could also play a role in pathogenesis in HD patients [36] and assays able to measure this fragment could be important.

To ensure that pre-clinical efficacy studies (e.g., knock-down treatments of the mutant HTT protein in HD animal models) are adequately powered to detect the predicted change in expression levels using the expanded polyglutamine MSD assay, we have performed a statistical power analysis of the assay for BAC HD, zQ175 KI and R6/2 HD mouse models (Figure S6). The estimation of the group size was carried out for 25% and 50% reduction in mutant HTT levels. We found that a maximum sample size of three animals is required to have a 90% probability of measuring a significant effect (difference between treated and untreated groups), distributed around the 25% true difference, using the expanded polyglutamine human HTT assay. The group size is reduced to two animals for a 50% effect (Table S2 in Text S1). The group size was comparable among the various HD mouse models tested, independently from the expression of full-length (BAC HD and zQ175 KI) or exon-1 (R6/2) human mutant HTT protein (Table S2 in Text S1).

Endogenous mouse HTT protein was successfully quantified in HD mouse models using the mouse HTT purified standard protein in the mouse HTT-specific MSD assay. The availability of a mouse HTT-specific assay, allowing the analysis of endogenous mouse HTT protein, provides, for the first time, the opportunity to accurately monitor the effects of therapeutic agents on wild type rodent HTT in pre-clinical studies.

Our human HTT bioassays allow an accurate detection of HTT in lysates obtained from human post mortem brain tissues as well as lymphoblast cell lines derived from both HD and non-HD control patients (Figure 6). Analysis of post mortem cortex homogenates of HD patients and non-HD control donors using the MSD assays for total and polyglutamine-expanded HTT showed comparable amounts of total HTT across all tested samples, with an approximate total average of 145 femtomoles HTT per milligram of total input protein. This suggests that total HTT levels are not affected by HD. Determined levels of both total and polyglutamine-expanded HTT are in accordance with previously published data using a HTT-specific TR-FRET assay [16]. Total and polyglutamine-expanded HTT protein levels in HD and non-HD control patients were also comparable between lymphoblast and brain tissue samples.

In addition, when tested in the expanded-polyglutamine HTT assay, HD patient-derived samples were clearly distinct from control patient samples, indicating that the HTT MSD assays have the potential to become a simple test to monitor the patients' response to novel HTT suppression therapeutics, making clinical development of such treatments more efficient. This also potentially enables phenotypic screening strategies for the discovery of novel approaches to allele-selective HTT lowering. The ability to measure HTT proteins in peripheral blood cells allows the incorporation of these endpoints in clinical trials aimed at lowering HTT via the modulation of cellular mechanisms implicated in disease pathogenesis, such as autophagy, the heat shock response, or the modulation of enzymes implicated in HTT post-translational modifications, which affect its turnover and degradation pathways.

It is also important to test these assays for their potential to monitor disease progression. We are currently testing the ability of these assays to monitor human HTT proteins in more accessible rodent and human tissue samples, such as whole blood and cerebrospinal fluid, in order to generate flexible assay protocols for HTT protein bioanalysis to aid pre-clinical and clinical drug development. Future assay development for HTT detection includes assays to monitor truncation or cleavage of HTT [37] and post-translational modifications as phosphorylation [38], sumoylation [39], ubiquitination [40] and acetylation [41], which have all been reported to modulate HTT-mediated neurotoxicity.

These MSD HTT protein quantitation bioassays represent an initial set of tools to define fundamental aspects of the biology of the HTT protein and a critical step forward for the field of HD research which will help facilitate the advancement of potential therapies for HD. Furthermore, we are committed to making these assays available to the wider HD research community as soon as is feasible.

## Supporting Information

Figure S1 **Determination of species-selectivity of MSD assays using species-specific peptides.** Competition between the peptides CHDI-90000208 (antigenic peptide of the pAb146 antibody), CHDI-90000209 (antigenic peptide of the pAb147 antibody) and CHDI-90000210 (unrelated HTT peptide included as a negative control) and human HTT (1-573) Q23 or mouse HTT (1–549) Q7 for binding to the capture antibody demonstrated species-specificity of pAb146 (A) and pAb147 (B).
(TIF)

Figure S2 **Detection of endogenous rat HTT protein.** Endogenous rat HTT expressed in RccHan∶WIST wild type rat whole brains was detected using the pAb147-MAB2166 antibody pair originally used to develop the mouse HTT MSD assay. The target epitope sequence of both antibodies used in this MSD assay is conserved between mouse and rat HTT proteins and rat endogenous HTT protein was, as expected, significantly detected. Homogenates generated from human-derived cortical tissue were included as a negative control and, as expected, showed signals at background level. Data are averages of n = 2 independent samples with correspondent standard deviations.
(TIF)

Figure S3 **Effect of the biological sample matrix on the HTT MSD assays.** A spiking-and-recovery experiment was performed and purified large fragment and full length HTT proteins were used to assess the effect of the biological sample matrix on the expanded polyglutamine human HTT MSD assay (antibody pair pAb146/MW1) (A), the pan (antibody pair pAb146/MAB2166) (B), and the exon-1 - pan (antibody pair pAb146/pAb137) (C) human HTT MSD assays. The effect of the sample biological matrix on signal recovery of the mouse HTT (1–549) Q7 protein in the mouse/rat HTT MSD assay (antibody pair pAb147/MAB2166) (D) was also tested. The indicated recombinant protein concentrations were spiked in 20 µg of (CBA×C57Bl/6) F1 (CBF) (B6CBAF1/OlaHsd, Harlan Olac) ‘wild type’ mouse brain homogenate (lysate) or in MSD assay buffer 1 (buffer). The mouse HTT (1–549) Q7 protein was spiked in 20 µg of a 3 month-old homozygous zQ175 mouse, carrying two chimeric mouse/human exon1 alleles. Mouse brain extracts were generated using the MSD assay buffer 1. Data are averages of n = 2 technical replicates with correspondent standard deviations.
(TIF)

Figure S4 **MSD assay performance with HTT purified proteins spiked in MSD assay buffer 2.** A (CBA×C57Bl/6) F1 (CBF) (B6CBAF1/OlaHsd, Harlan Olac) ‘wild type’ mouse brain homogenate was generated using an alternative lysis buffer (MSD assay buffer 2: 50 mM Tris, pH 7.4, 120 mM NaCl, 0.5% NP-40, 1 mM EDTA, 1 mM DTT, 1 mM PMSF, protease inhibitors (Complete, EDTA-free; Roche Diagnostics)) and used in a spike-and-recovery experiment with the HTT (1–573) Q23 and HTT (1–573) Q73 large fragment proteins. MSD signals obtained for the different HTT proteins spiked in 20 µg of wild type mouse brain homogenate (lysate) or in MSD assay buffer 2 (buffer) in the expanded polyglutamine human HTT MSD assay (antibody pair pAb146/MW1) (A) and in the exon-1 - pan human HTT MSD assay (antibody pair pAb146/pAb137) (B) are shown. Data are averages of n = 2 technical replicates with correspondent standard deviations.
(TIF)

Figure S5 **Decrease of soluble mutant HTT levels in dissected R6/2 brain tissues is associated to increased age of the mice.** Homogenates from cortical (A), striatal (B), and brain stem (C) regions from R6/2 female mice (bearing an expanded polyglutamine tract of 206 CAG repeats on average) were analyzed for detection of soluble mutant human HTT at different ages (4, 8, 12 and 15 weeks). All brain regions analyzed showed significant signals in the expanded polyglutamine human HTT MSD assay (antibody pair pAb146/MW1) and a progressive signal decrease over time. Data are averages of n = 3 independent samples with correspondent standard deviations. B, assay background. *, P<0.05; **, P<0.01; ***, P<0.001.
(TIF)

Figure S6 **Group size estimation for sample analysis in the expanded polyglutamine human HTT MSD assay.** Expanded polyglutamine human HTT MSD assay power analysis was performed for BAC HD (A), zQ175 KI (B) and R6/2 (C) HD mouse models. Brain extracts generated from different HD mice were mixed at different ratios with a brain homogenate generated from one correspondent wild type mouse (HD∶WT ratios 100∶0, 75∶0 and 50∶50). The so prepared samples were tested in the expanded polyglutamine human HTT MSD assay (antibody pair pAb146/MW1). Electrochemiluminescence signals obtained for each sample tested are represented by the different histograms. Tables show the inter-samples variability, the average residual MSD signal and the average MSD signal reduction for the different HD∶WT ratios and for the different HD mouse models. Data are averages of n = 3 technical replicates with correspondent standard deviations. The coefficient of variation (CV) is defined as the ratio of the standard deviation to the mean.
(TIF)

Figure S7 **Production of purified human HTT proteins.** (A) SDS-PAGE of FLAG affinity purified HTT (1–573) Q23 and HTT (1–573) Q73 proteins. e1–e5 represent each 1 ml elution with the FLAG peptide. M, molecular weight marker (kDa). Additional proteins/truncated products are visible on the gel. (B) SDS-PAGE of Superdex 200 16/60 gel filtration column-purified HTT (1–573) Q23 and HTT (1–573) Q73 proteins. A dilution series from 1,600 ng to 100 ng of both HTT (1–573) Q23 (lane 1–5) and HTT (1–573) Q73 (lane 6–10) proteins was loaded on the gel. M, molecular weight marker (kDa). The concentrations of both proteins were determined by Bradford assay, in triplicate.
(TIF)

Figure S8 **Determination of human HTT (1–573) Q23 and HTT (1–573) Q73 recombinant protein stability.** (A) Native gel electrophoresis of purified proteins in 50 mM Tris pH 7.4, 500 mM NaCl, 10% glycerol, 0.1% CHAPS, 1 mM EDTA (lane 1) or in the same buffer with the following modifications: 50% glycerol (lane 2), 0.25% BSA (lane 3), 1 M sodium chloride (lane 4), 25% glycerol, 0.25% BSA and 1 M sodium chloride (lane 5) after 72 h or 57 days of storage at −80°C. Monomeric HTT (1–573) Q23 and HTT (1–573) Q73 are indicated by arrows. C, 0.25% BSA only. M, molecular weight marker (kDa). (B) Native gel analysis of purified HTT (1–573) Q23 and HTT (1–573) Q73 large fragment proteins stored in 50 mM Tris pH 7.4, 500 mM NaCl, 10% glycerol, 0.1% CHAPS, 1 mM EDTA after 386 days of storage at −80°C. Monomeric and higher molecular weight HTT (1–573) Q23 and HTT (1–573) Q73 are indicated by arrows. M, molecular weight marker (kDa).
(TIF)

Figure S9 **Production of purified mouse HTT protein.** (A) SDS-PAGE of FLAG affinity purified mouse HTT (1–549) Q7 protein. Cells from 3 L culture were lysed by freeze/thaw in a buffer containing 50 mM Tris pH 7.4, 500 mM NaCl, 10% glycerol, 1% CHAPS, 1 mM EDTA and Complete EDTA-free protease inhibitors. Soluble fractions following centrifugation were incubated with anti-FLAG M2 affinity gel overnight at 4°C before washing (w1–w3) and eluting with 5×1 ml of 0.4 mg/ml FLAG peptide in 50 mM Tris pH 7.4, 500 mM NaCl, 10% glycerol, 1% CHAPS, 1 mM EDTA (e1–e5). M, molecular weight marker (kDa). (B) SDS-PAGE of Superdex 200 16/60 purified mouse HTT (1–549) Q7 protein. M, molecular weight marker (kDa). Ld, sample loaded onto the column. (C) MonoQ 5/50 chromatogram showing ion exchange separation of mouse HTT (1–549) Q7 protein. The mouse HTT (1–549) Q7 protein eluted as two distinct peaks in the middle of the NaCl gradient. (D) SDS-PAGE of MonoQ 5/50 ion exchange purified mouse HTT (1–549) Q7 protein. M, molecular weight marker (kDa). Ld, sample loaded onto the column. Protein contained in each peak was separately pooled and concentrated. Protein concentration was determined by Bradford assay, in triplicate.
(TIF)

Text S1 (DOCX)

## References

[1] The Huntington's Disease Collaborative Research Group (1993) A novel gene containing a trinucleotide repeat that is expanded and unstable on Huntington's disease chromosomes. Cell 72: 971–983. 8458085 10.1016/0092-8674(93)90585-e

[2] Sharp AH, Loev SJ, Schilling G, Li SH, Li XJ, et al (1995) Widespread expression of Huntington's disease gene (IT15) protein product. Neuron 14: 1065–1074. 7748554 10.1016/0896-6273(95)90345-3

[3] Tabrizi SJ, Langbehn DR, Leavitt BR, Roos RA, Durr A, et al (2009) Biological and clinical manifestations of Huntington's disease in the longitudinal TRACK-HD study: cross-sectional analysis of baseline data. Lancet Neurol 8: 791–801. 19646924 10.1016/S1474-4422(09)70170-X PMC3725974

[4] Carroll JB, Warby SC, Southwell AL, Doty CN, Greenlee S, et al (2011) Potent and selective antisense oligonucleotides targeting single-nucleotide polymorphisms in the huntington disease gene/allele-specific silencing of mutant huntingtin. Mol Ther 19: 2178–2185. 21971427 10.1038/mt.2011.201 PMC3242664

[5] McBride JL, Pitzer MR, Boudreau RL, Dufour B, Hobbs T, et al (2011) Preclinical Safety of RNAi-Mediated HTT Suppression in the Rhesus Macaque as a Potential Therapy for Huntington's Disease. Mol Ther 19: 2152–2162. 22031240 10.1038/mt.2011.219 PMC3242667

[6] Sah DW, Aronin N (2011) Oligonucleotide therapeutic approaches for Huntington disease. J Clin Invest 121: 500–507. 21285523 10.1172/JCI45130 PMC3026739

[7] Hu J, Matsui M, Gagnon KT, Schwartz JC, Gabillet S, et al (2009) Allele-specific silencing of mutant huntingtin and ataxin-3 genes by targeting expanded CAG repeats in mRNAs. Nat Biotechnol 27: 478–484. 19412185 10.1038/nbt.1539 PMC2765218

[8] Harper SQ, Staber PD, He X, Eliason SL, Martins IH, et al (2005) RNA interference improves motor and neuropathological abnormalities in a Huntington's disease mouse model. Proc Natl Acad Sci U S A 102: 5820–5825. 15811941 10.1073/pnas.0501507102 PMC556303

[9] DiFiglia M, Sena-Esteves M, Chase K, Sapp E, Pfister E, et al (2007) Therapeutic silencing of mutant huntingtin with siRNA attenuates striatal and cortical neuropathology and behavioral deficits. Proc Natl Acad Sci U S A 104: 17204–17209. 17940007 10.1073/pnas.0708285104 PMC2040405

[10] Kordasiewicz HB, Stanek LM, Wancewicz EV, Mazur C, McAlonis MM, et al (2012) Sustained Therapeutic Reversal of Huntington's Disease by Transient Repression of Huntingtin Synthesis. Neuron 74: 1031–1044. 22726834 10.1016/j.neuron.2012.05.009 PMC3383626

[11] Stanek LM, Yang W, Angus S, Sardi PS, Hayden MR, et al (2013) Antisense Oligonucleotide-Mediated Correction of Transcriptional Dysregulation is Correlated with Behavioral Benefits in the YAC128 Mouse Model of Huntington's Disease. J Huntington's Disease 2: 217–228. 25063516 10.3233/JHD-130057

[12] DiFiglia M, Sapp E, Chase KO, Davies SW, Bates GP, et al (1997) Aggregation of huntingtin in neuronal intranuclear inclusions and dystrophic neurites in brain. Science 277: 1990–1993. 9302293 10.1126/science.277.5334.1990

[13] Miller J, Arrasate M, Brooks E, Libeu CP, Legleiter J, et al (2011) Identifying polyglutamine protein species in situ that best predict neurodegeneration. Nat Chem Biol 7: 925–934. 22037470 10.1038/nchembio.694 PMC3271120

[14] Pyati R, Richter MM (2007) ECL–Electrochemical luminescence. Annu Rep Prog Chem, Sect C 103: 12–78.

[15] Fu Q, Zhu J, Van Eyk JE (2010) Comparison of multiplex immunoassay platforms. Clin Chem 56: 314–318. 20022982 10.1373/clinchem.2009.135087 PMC2905867

[16] Weiss A, Abramowski D, Bibel M, Bodner R, Chopra V, et al (2009) Single-step detection of mutant huntingtin in animal and human tissues: A bioassay for Huntington's disease. Anal Biochem 395: 8–15. 19664996 10.1016/j.ab.2009.08.001

[17] Paganetti P, Weiss A, Trapp M, Hammerl I, Bleckmann D, et al (2009) Development of a method for the high-throughput quantification of cellular proteins. Chembiochem 10: 1678–1688. 19492395 10.1002/cbic.200900131

[18] Sathasivam K, Lane A, Legleiter J, Warley A, Woodman B, et al (2010) Identical oligomeric and fibrillar structures captured from the brains of R6/2 and knock-in mouse models of Huntington's disease. Hum Mol Genet 19: 65–78. 19825844 10.1093/hmg/ddp467 PMC2792149

[19] Davies SW, Sathasivam K, Hobbs C, Doherty P, Mangiarini L, et al (1999) Detection of polyglutamine aggregation in mouse models. Methods Enzymol 309: 687–701. 10507055 10.1016/s0076-6879(99)09045-x

[20] Ko J, Ou S, Patterson PH (2001) New anti-huntingtin monoclonal antibodies: implications for huntingtin conformation and its binding proteins. Brain Res Bull 56: 319–329. 11719267 10.1016/s0361-9230(01)00599-8

[21] Duzdevich D, Li J, Whang J, Takahashi H, Takeyasu K, et al (2011) Unusual Structures Are Present in DNA Fragments Containing Super-Long Huntingtin CAG Repeats. PLoS ONE 6: e17119. 21347256 10.1371/journal.pone.0017119 PMC3037965

[22] Mangiarini L, Sathasivam K, Seller M, Cozens B, Harper A, et al (1996) Exon 1 of the HD gene with an expanded CAG repeat is sufficient to cause a progressive neurological phenotype in transgenic mice. Cell 87: 493–506. 8898202 10.1016/s0092-8674(00)81369-0

[23] Gray M, Shirasaki DI, Cepeda C, Andre VM, Wilburn B, et al (2008) Full-length human mutant huntingtin with a stable polyglutamine repeat can elicit progressive and selective neuropathogenesis in BACHD mice. J Neurosci 28: 6182–6195. 18550760 10.1523/JNEUROSCI.0857-08.2008 PMC2630800

[24] Menalled LB, Sison JD, Dragatsis I, Zeitlin S, Chesselet MF (2003) Time course of early motor and neuropathological anomalies in a knock-in mouse model of Huntington's disease with 140 CAG repeats. J Comp Neurol 465: 11–26. 12926013 10.1002/cne.10776

[25] Heikkinen T, Lehtimaki K, Vartiainen N, Puolivali J, Hendricks SJ, et al (2012) Characterization of neurophysiological and behavioral changes, MRI brain volumetry and 1H MRS in zQ175 knock-in mouse model of Huntington's disease. PLoS One 7: e50717. 23284644 10.1371/journal.pone.0050717 PMC3527436

[26] Menalled LB, Kudwa AE, Miller S, Fitzpatrick J, Watson-Johnson J, et al (2012) Comprehensive behavioral and molecular characterization of a new knock-in mouse model of Huntington's disease: zQ175. PLoS One 7: e49838. 23284626 10.1371/journal.pone.0049838 PMC3527464

[27] Kügler S, Lingor P, Scholl U, Zolotukhin S, Bahr M (2003) Differential transgene expression in brain cells in vivo and in vitro from AAV-2 vectors with small transcriptional control units. Virology 311: 89–95. 12832206 10.1016/s0042-6822(03)00162-4

[28] McBride JL, Boudreau RL, Harper SQ, Staber PD, Monteys AM, et al (2008) Artificial miRNAs mitigate shRNA-mediated toxicity in the brain: implications for the therapeutic development of RNAi. Proc Natl Acad Sci U S A 105: 5868–5873. 18398004 10.1073/pnas.0801775105 PMC2311380

[29] Huang B, Schiefer J, Sass C, Kosinski CM, Kochanek S (2008) Inducing huntingtin inclusion formation in primary neuronal cell culture and in vivo by high-capacity adenoviral vectors expressing truncated and full-length huntingtin with polyglutamine expansion. J Gene Med 10: 269–279. 18067195 10.1002/jgm.1150

[30] Cong SY, Pepers BA, Roos RA, Van Ommen GJ, Dorsman JC (2005) Epitope mapping of monoclonal antibody 4C8 recognizing the protein huntingtin. Hybridoma (Larchmt) 24: 231–235. 16225422 10.1089/hyb.2005.24.231

[31] Bates G (2003) Huntingtin aggregation and toxicity in Huntington's disease. Lancet 361: 1642–1644. 12747895 10.1016/S0140-6736(03)13304-1

[32] Li P, Huey-Tubman KE, Gao T, Li X, West AP Jr, et al (2007) The structure of a polyQ-anti-polyQ complex reveals binding according to a linear lattice model. Nat Struct Mol Biol 14: 381–387. 17450152 10.1038/nsmb1234

[33] Baldo B, Paganetti P, Grueninger S, Marcellin D, Kaltenbach LS, et al (2012) TR-FRET-Based Duplex Immunoassay Reveals an Inverse Correlation of Soluble and Aggregated Mutant huntingtin in Huntington's Disease. Chem Biol 19: 264–275. 22365609 10.1016/j.chembiol.2011.12.020

[34] Scherzinger E, Sittler A, Schweiger K, Heiser V, Lurz R, et al (1999) Self-assembly of polyglutamine-containing huntingtin fragments into amyloid-like fibrils: Implications for Huntington's disease pathology. Proc Natl Acad Sci USA 96: 4604–4609. 10200309 10.1073/pnas.96.8.4604 PMC16379

[35] Weiss A, Trager U, Wild EJ, Grueninger S, Farmer R, et al (2012) Mutant huntingtin fragmentation in immune cells tracks Huntington's disease progression. J Clin Invest 122: 3731–3736. 22996692 10.1172/JCI64565 PMC3461928

[36] Sathasivam K, Neueder A, Gipson TA, Landles C, Benjamin AC, et al (2013) Aberrant splicing of HTT generates the pathogenic exon 1 protein in Huntington disease. Proc Natl Acad Sci U S A 110: 2366–2370. 23341618 10.1073/pnas.1221891110 PMC3568346

[37] Graham RK, Deng Y, Slow EJ, Haigh B, Bissada N, et al (2006) Cleavage at the caspase-6 site is required for neuronal dysfunction and degeneration due to mutant huntingtin. Cell 125: 1179–1191. 16777606 10.1016/j.cell.2006.04.026

[38] Pardo R, Colin E, Regulier E, Aebischer P, Deglon N, et al (2006) Inhibition of calcineurin by FK506 protects against polyglutamine-huntingtin toxicity through an increase of huntingtin phosphorylation at S421. J Neurosci 26: 1635–1645. 16452687 10.1523/JNEUROSCI.3706-05.2006 PMC6675484

[39] Steffan JS, Agrawal N, Pallos J, Rockabrand E, Trotman LC, et al (2004) SUMO modification of Huntingtin and Huntington's disease pathology. Science 304: 100–104. 15064418 10.1126/science.1092194

[40] Kalchman MA, Graham RK, Xia G, Koide HB, Hodgson JG, et al (1996) Huntingtin is ubiquitinated and interacts with a specific ubiquitin- conjugating enzyme. J Biol Chem 271: 19385–19394. 8702625 10.1074/jbc.271.32.19385

[41] Jeong H, Then F, Melia TJ Jr, Mazzulli JR, Cui L, et al (2009) Acetylation targets mutant huntingtin to autophagosomes for degradation. Cell 137: 60–72. 19345187 10.1016/j.cell.2009.03.018 PMC2940108

[42] Zhao Y, Chapman DA, Jones IM (2003) Improving baculovirus recombination. Nucleic Acids Res. 31(2): E6–6. 10.1093/nar/gng006 PMC140531 12527795

