# Supplementary figures and images for: Quantification Assays for Total and Polyglutamine-Expanded Huntingtin Proteins
Source: PLoS One. 2014 May 9;9(5):e96854. doi: 10.1371/journal.pone.0096854 (PMC4016121; doi:10.1371/journal.pone.0096854)

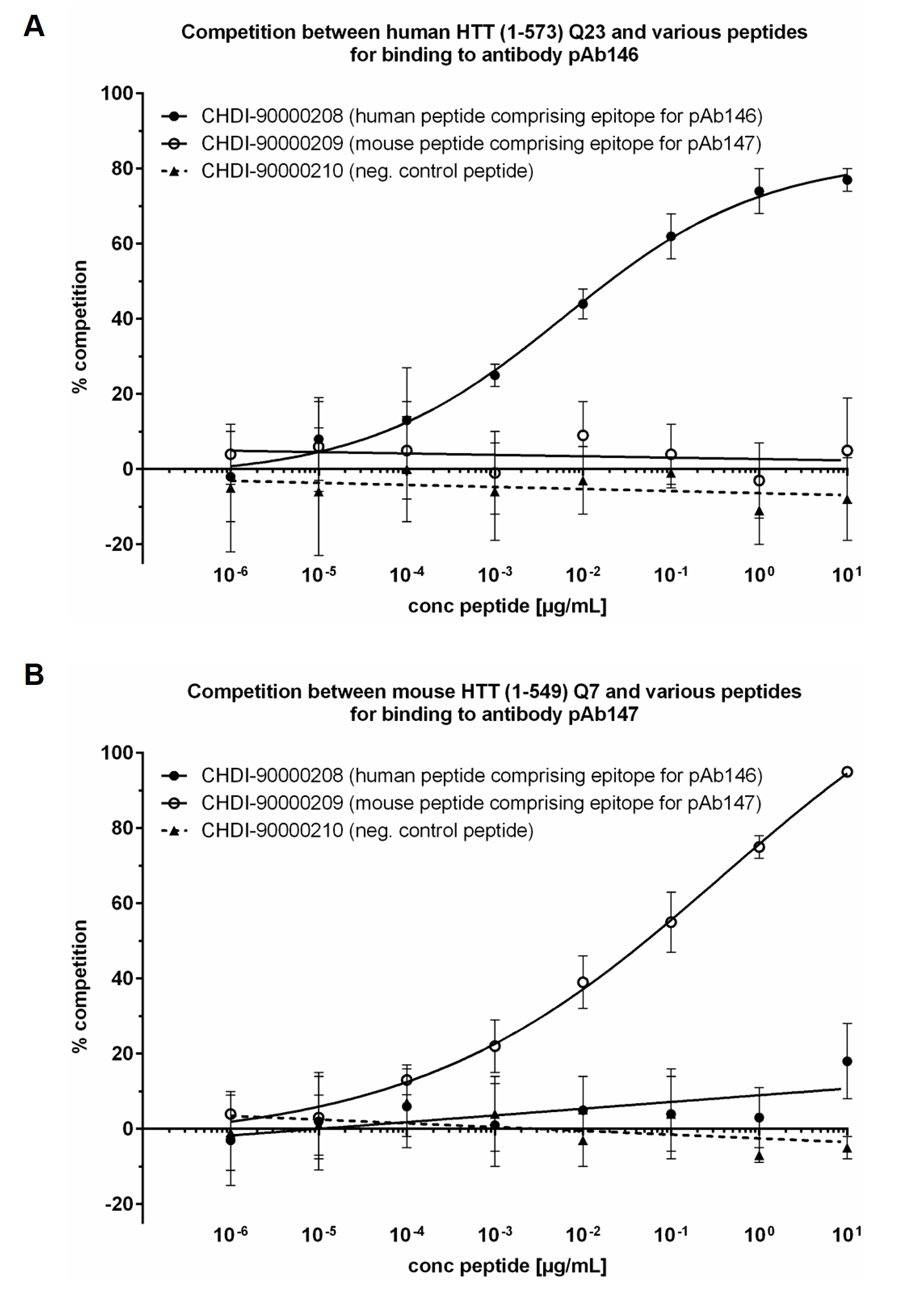

Supplement: Figure S1 — Determination of species-selectivity of MSD assays using species-specific peptides. Competition between the peptides CHDI-90000208 (antigenic peptide of the pAb146 antibody), CHDI-90000209 (antigenic peptide of the pAb147 antibody) and CHDI-90000210 (unrelated HTT peptide included as a negative control) and human HTT (1-573) Q23 or mouse HTT (1–549) Q7 for binding to the capture antibody demonstrated species-specificity of pAb146 (A) and pAb147 (B). (TIF) [file pone.0096854.s001.tif]

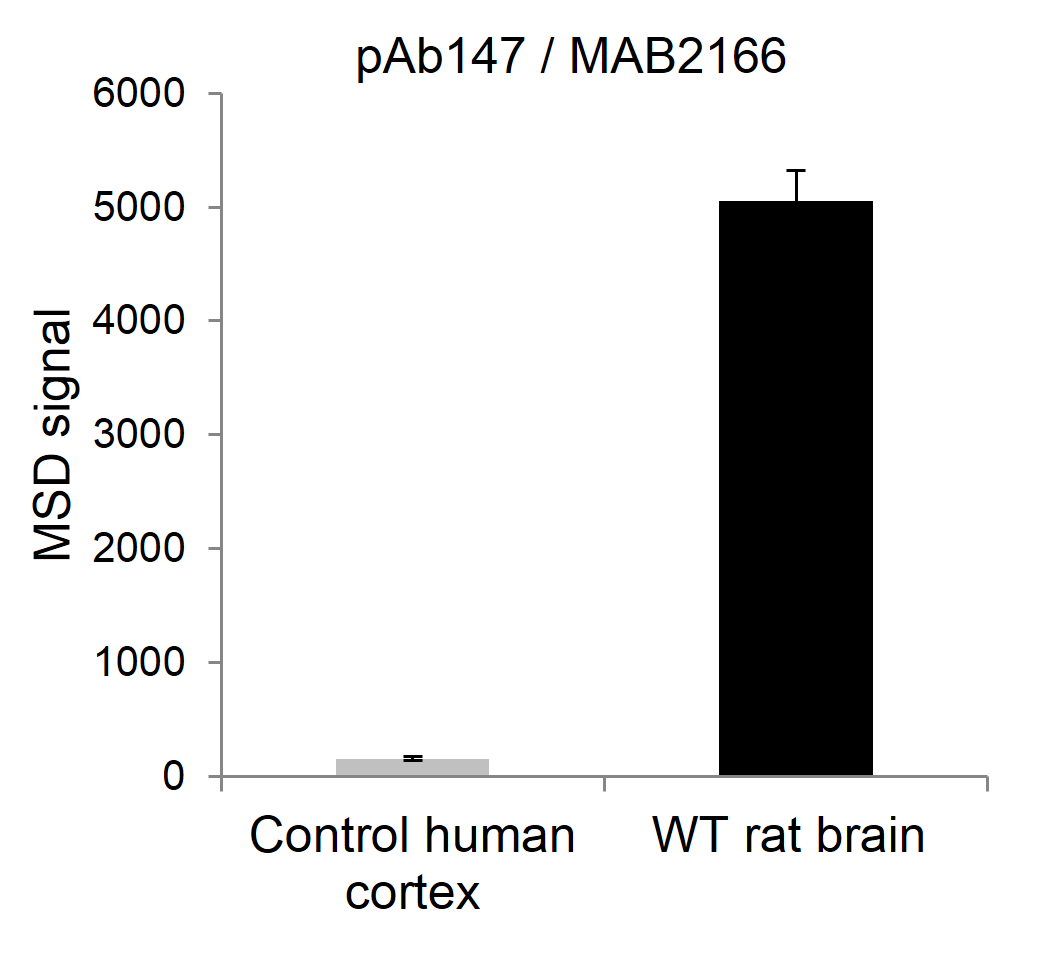

Supplement: Figure S2 — Detection of endogenous rat HTT protein. Endogenous rat HTT expressed in RccHan∶WIST wild type rat whole brains was detected using the pAb147-MAB2166 antibody pair originally used to develop the mouse HTT MSD assay. The target epitope sequence of both antibodies used in this MSD assay is conserved between mouse and rat HTT proteins and rat endogenous HTT protein was, as expected, significantly detected. Homogenates generated from human-derived cortical tissue were included as a negative control and, as expected, showed signals at background level. Data are averages of n = 2 independent samples with correspondent standard deviations. (TIF) [file pone.0096854.s002.tif]

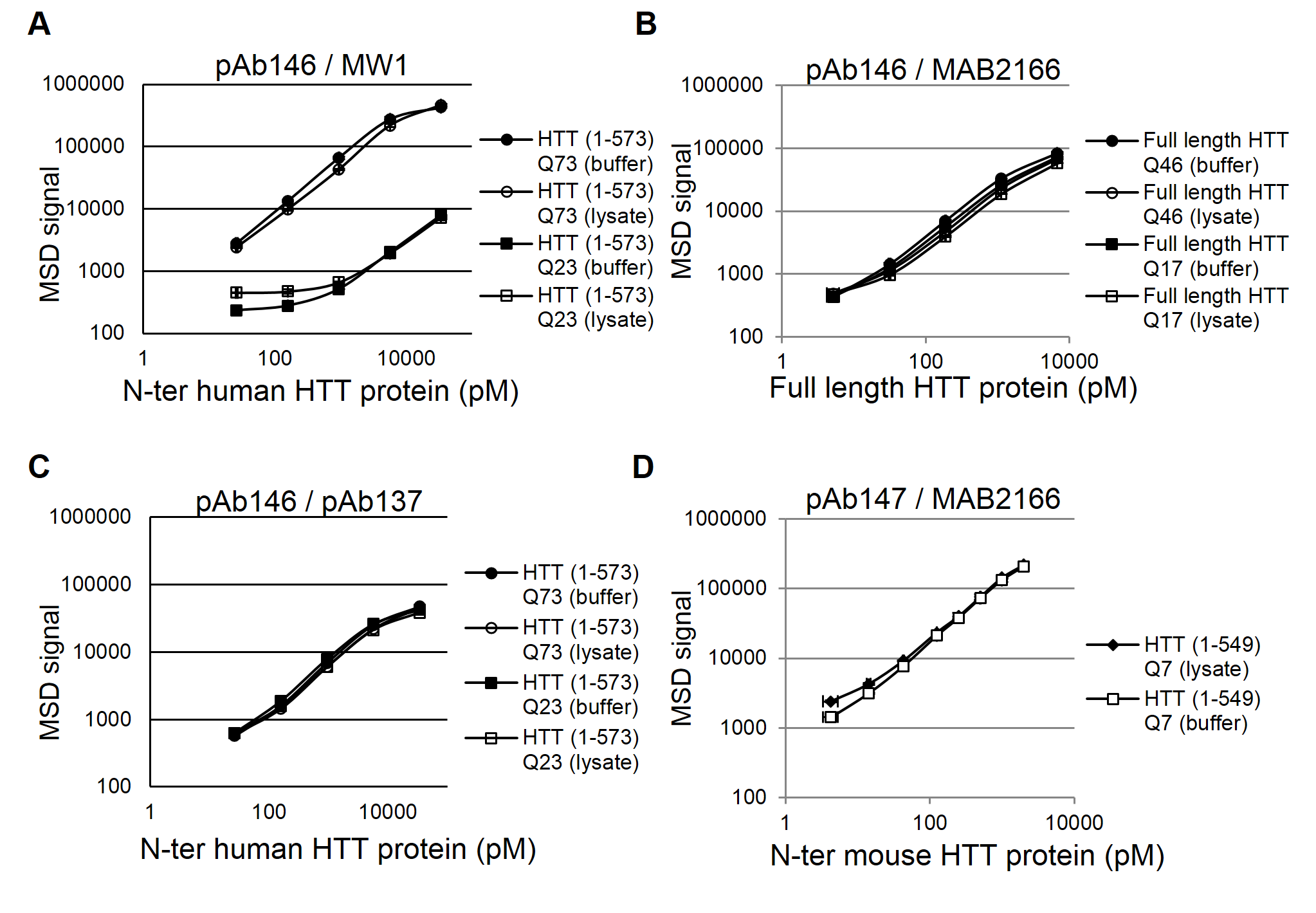

Supplement: Figure S3 — Effect of the biological sample matrix on the HTT MSD assays. A spiking-and-recovery experiment was performed and purified large fragment and full length HTT proteins were used to assess the effect of the biological sample matrix on the expanded polyglutamine human HTT MSD assay (antibody pair pAb146/MW1) (A), the pan (antibody pair pAb146/MAB2166) (B), and the exon-1 - pan (antibody pair pAb146/pAb137) (C) human HTT MSD assays. The effect of the sample biological matrix on signal recovery of the mouse HTT (1–549) Q7 protein in the mouse/rat HTT MSD assay (antibody pair pAb147/MAB2166) (D) was also tested. The indicated recombinant protein concentrations were spiked in 20 µg of (CBA×C57Bl/6) F1 (CBF) (B6CBAF1/OlaHsd, Harlan Olac) ‘wild type’ mouse brain homogenate (lysate) or in MSD assay buffer 1 (buffer). The mouse HTT (1–549) Q7 protein was spiked in 20 µg of a 3 month-old homozygous zQ175 mouse, carrying two chimeric mouse/human exon1 alleles. Mouse brain extracts were generated using the MSD assay buffer 1. Data are averages of n = 2 technical replicates with correspondent standard deviations. (TIF) [file pone.0096854.s003.tif]

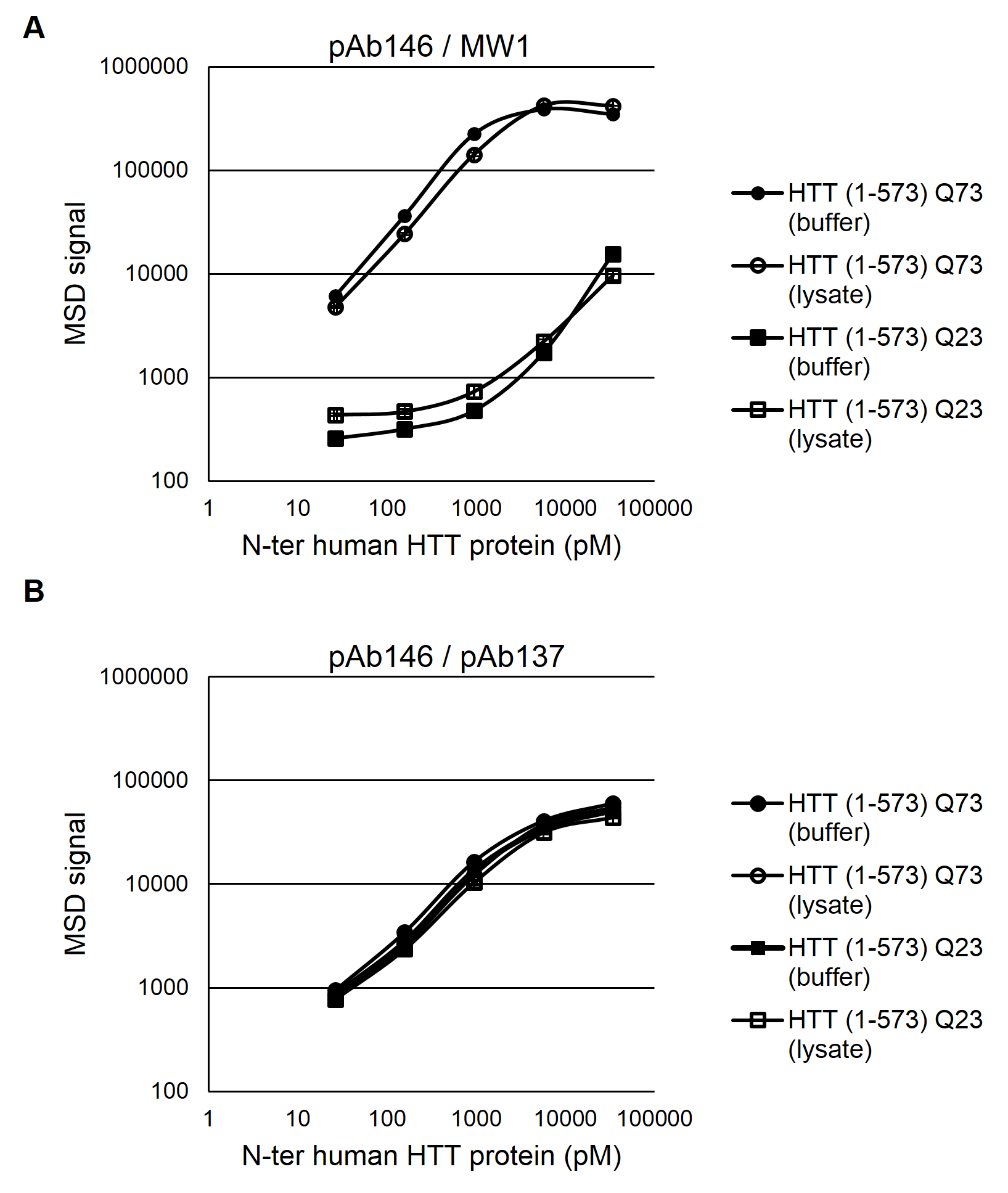

Supplement: Figure S4 — MSD assay performance with HTT purified proteins spiked in MSD assay buffer 2. A (CBA×C57Bl/6) F1 (CBF) (B6CBAF1/OlaHsd, Harlan Olac) ‘wild type’ mouse brain homogenate was generated using an alternative lysis buffer (MSD assay buffer 2: 50 mM Tris, pH 7.4, 120 mM NaCl, 0.5% NP-40, 1 mM EDTA, 1 mM DTT, 1 mM PMSF, protease inhibitors (Complete, EDTA-free; Roche Diagnostics)) and used in a spike-and-recovery experiment with the HTT (1–573) Q23 and HTT (1–573) Q73 large fragment proteins. MSD signals obtained for the different HTT proteins spiked in 20 µg of wild type mouse brain homogenate (lysate) or in MSD assay buffer 2 (buffer) in the expanded polyglutamine human HTT MSD assay (antibody pair pAb146/MW1) (A) and in the exon-1 - pan human HTT MSD assay (antibody pair pAb146/pAb137) (B) are shown. Data are averages of n = 2 technical replicates with correspondent standard deviations. (TIF) [file pone.0096854.s004.tif]

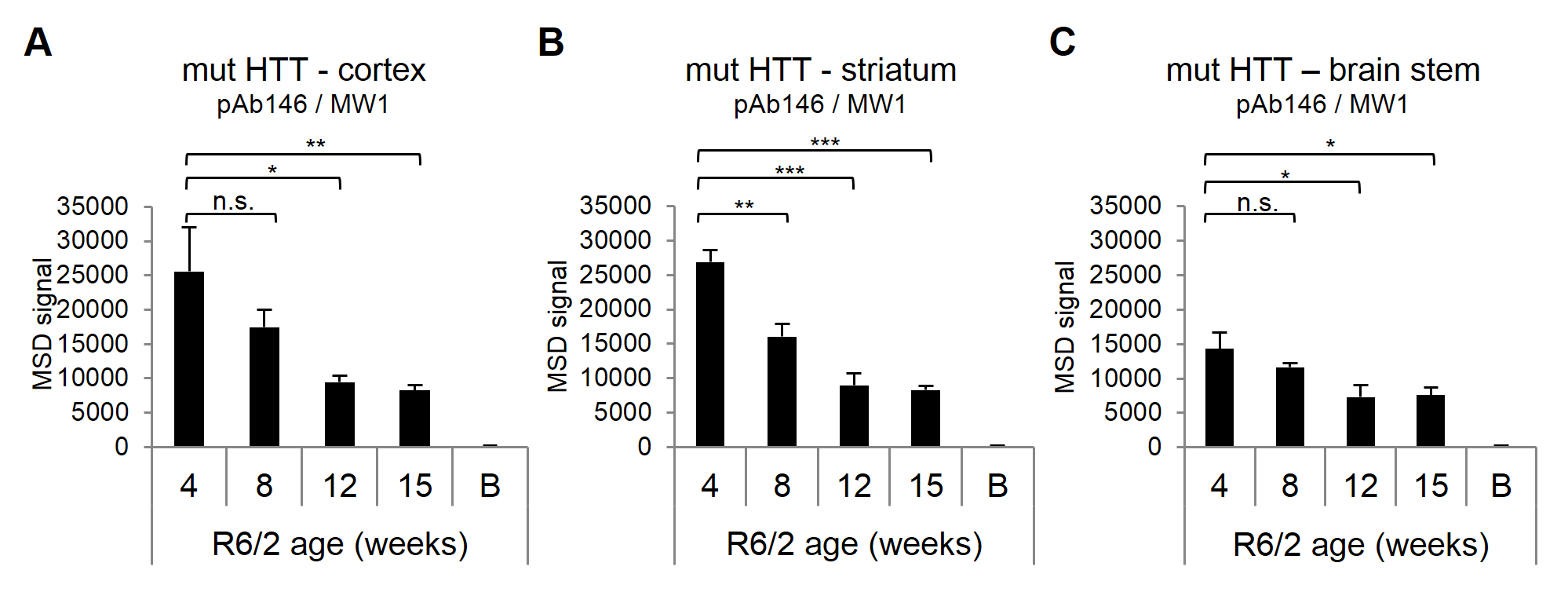

Supplement: Figure S5 — Decrease of soluble mutant HTT levels in dissected R6/2 brain tissues is associated to increased age of the mice. Homogenates from cortical (A), striatal (B), and brain stem (C) regions from R6/2 female mice (bearing an expanded polyglutamine tract of 206 CAG repeats on average) were analyzed for detection of soluble mutant human HTT at different ages (4, 8, 12 and 15 weeks). All brain regions analyzed showed significant signals in the expanded polyglutamine human HTT MSD assay (antibody pair pAb146/MW1) and a progressive signal decrease over time. Data are averages of n = 3 independent samples with correspondent standard deviations. B, assay background. *, P<0.05; **, P<0.01; ***, P<0.001. (TIF) [file pone.0096854.s005.tif]

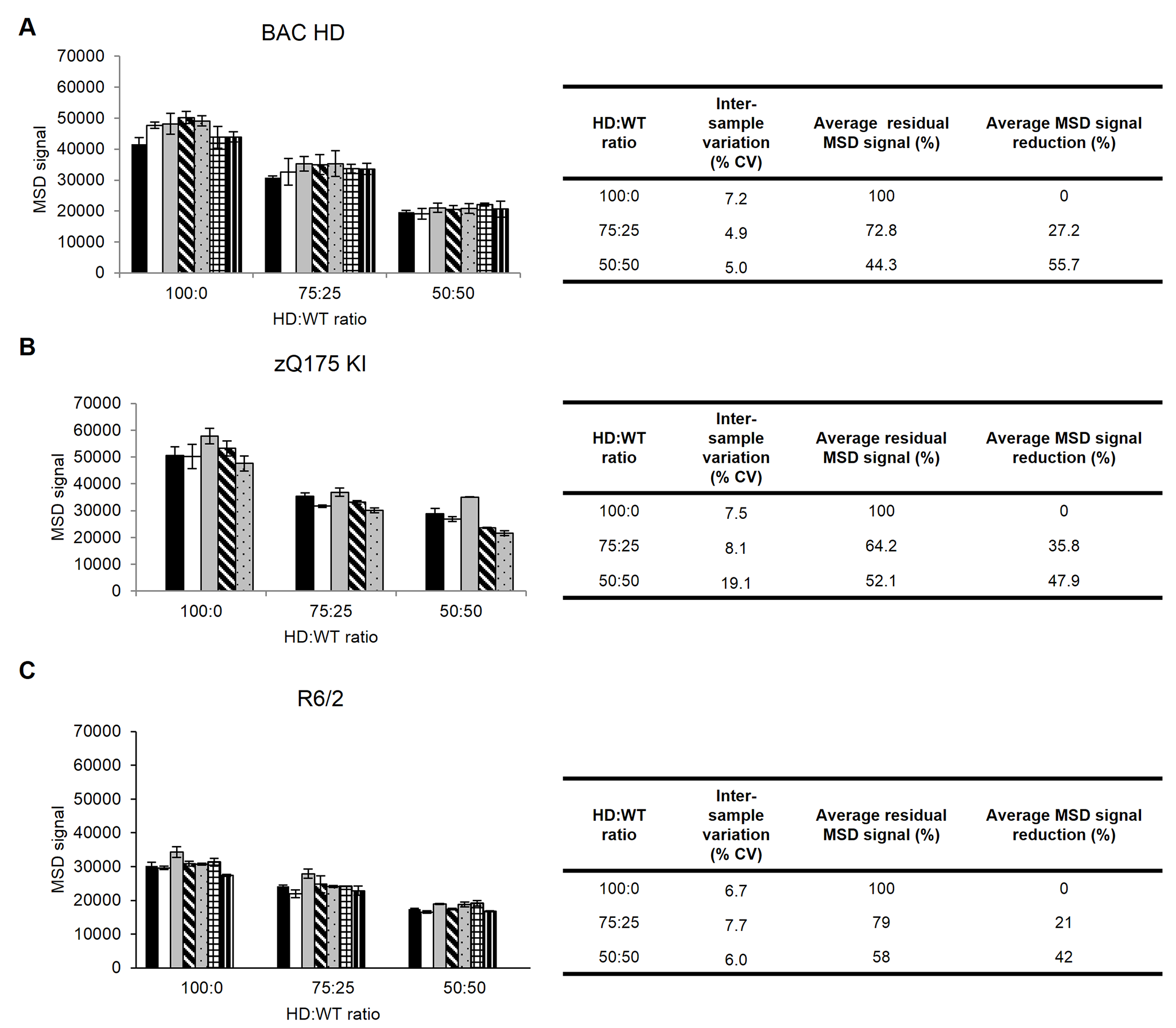

Supplement: Figure S6 — Group size estimation for sample analysis in the expanded polyglutamine human HTT MSD assay. Expanded polyglutamine human HTT MSD assay power analysis was performed for BAC HD (A), zQ175 KI (B) and R6/2 (C) HD mouse models. Brain extracts generated from different HD mice were mixed at different ratios with a brain homogenate generated from one correspondent wild type mouse (HD∶WT ratios 100∶0, 75∶0 and 50∶50). The so prepared samples were tested in the expanded polyglutamine human HTT MSD assay (antibody pair pAb146/MW1). Electrochemiluminescence signals obtained for each sample tested are represented by the different histograms. Tables show the inter-samples variability, the average residual MSD signal and the average MSD signal reduction for the different HD∶WT ratios and for the different HD mouse models. Data are averages of n = 3 technical replicates with correspondent standard deviations. The coefficient of variation (CV) is defined as the ratio of the standard deviation to the mean. (TIF) [file pone.0096854.s006.tif]

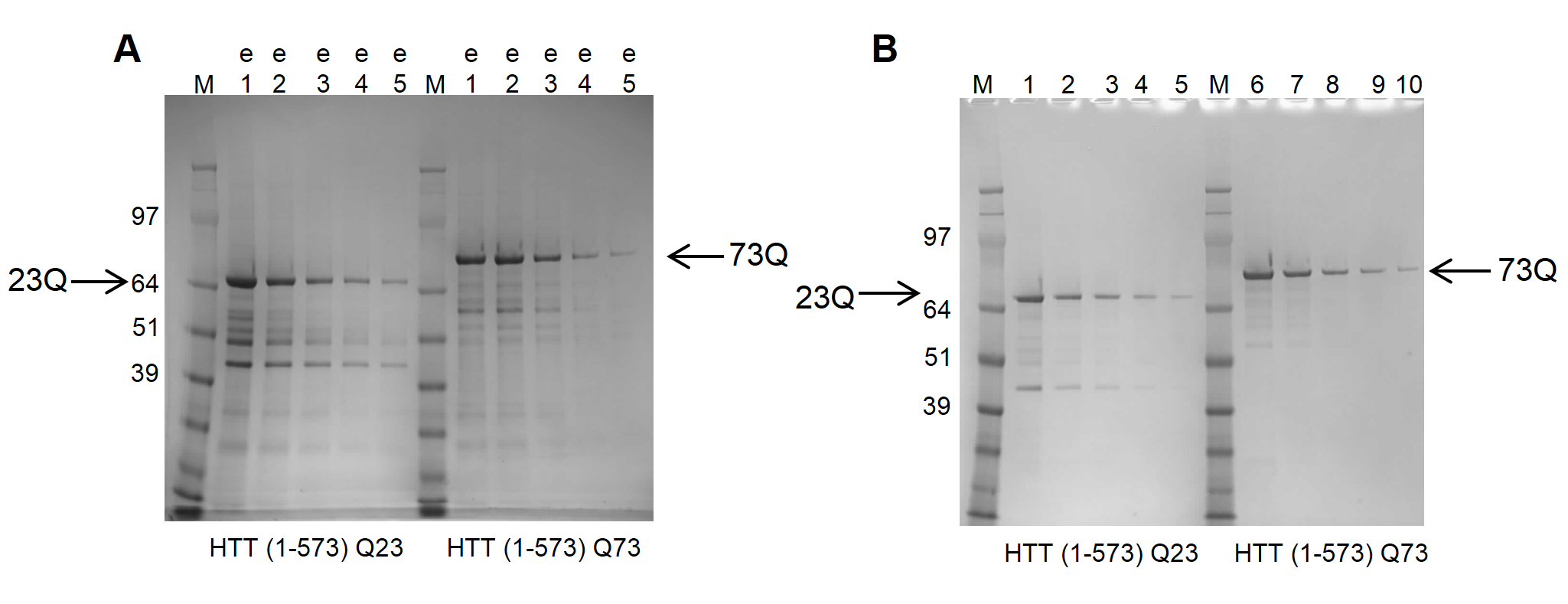

Supplement: Figure S7 — Production of purified human HTT proteins. (A) SDS-PAGE of FLAG affinity purified HTT (1–573) Q23 and HTT (1–573) Q73 proteins. e1–e5 represent each 1 ml elution with the FLAG peptide. M, molecular weight marker (kDa). Additional proteins/truncated products are visible on the gel. (B) SDS-PAGE of Superdex 200 16/60 gel filtration column-purified HTT (1–573) Q23 and HTT (1–573) Q73 proteins. A dilution series from 1,600 ng to 100 ng of both HTT (1–573) Q23 (lane 1–5) and HTT (1–573) Q73 (lane 6–10) proteins was loaded on the gel. M, molecular weight marker (kDa). The concentrations of both proteins were determined by Bradford assay, in triplicate. (TIF) [file pone.0096854.s007.tif]

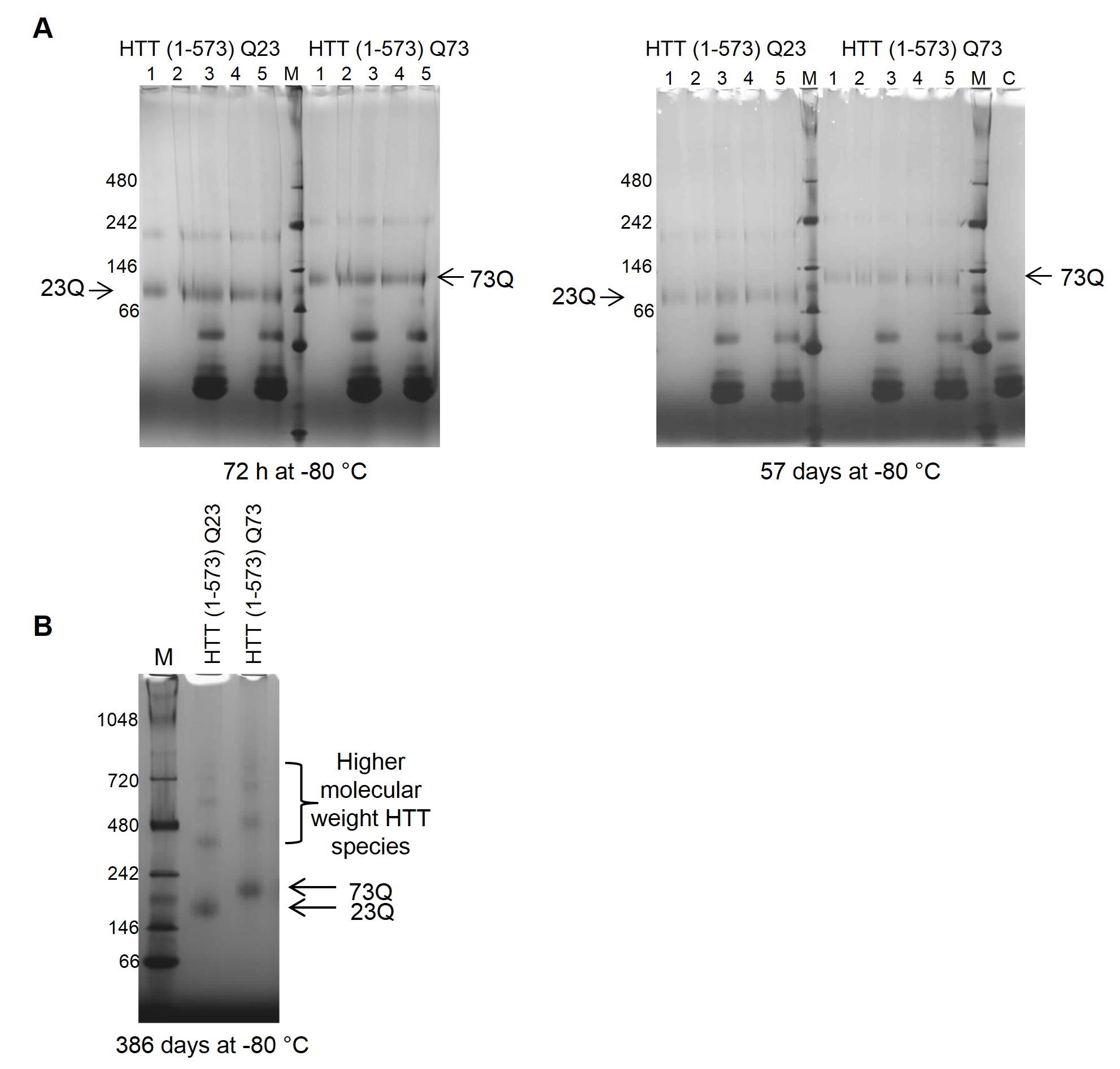

Supplement: Figure S8 — Determination of human HTT (1–573) Q23 and HTT (1–573) Q73 recombinant protein stability. (A) Native gel electrophoresis of purified proteins in 50 mM Tris pH 7.4, 500 mM NaCl, 10% glycerol, 0.1% CHAPS, 1 mM EDTA (lane 1) or in the same buffer with the following modifications: 50% glycerol (lane 2), 0.25% BSA (lane 3), 1 M sodium chloride (lane 4), 25% glycerol, 0.25% BSA and 1 M sodium chloride (lane 5) after 72 h or 57 days of storage at −80°C. Monomeric HTT (1–573) Q23 and HTT (1–573) Q73 are indicated by arrows. C, 0.25% BSA only. M, molecular weight marker (kDa). (B) Native gel analysis of purified HTT (1–573) Q23 and HTT (1–573) Q73 large fragment proteins stored in 50 mM Tris pH 7.4, 500 mM NaCl, 10% glycerol, 0.1% CHAPS, 1 mM EDTA after 386 days of storage at −80°C. Monomeric and higher molecular weight HTT (1–573) Q23 and HTT (1–573) Q73 are indicated by arrows. M, molecular weight marker (kDa). (TIF) [file pone.0096854.s008.tif]

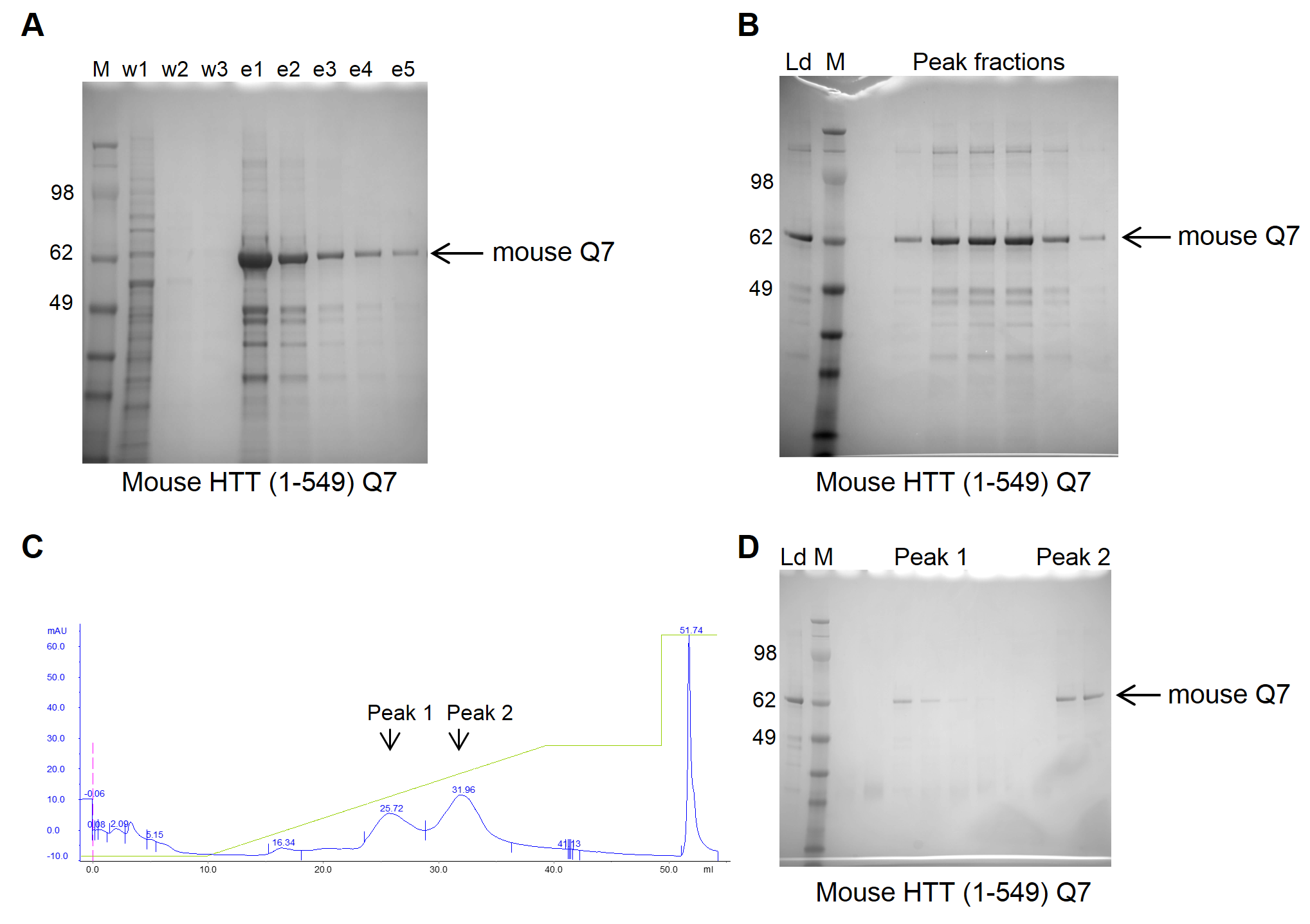

Supplement: Figure S9 — Production of purified mouse HTT protein. (A) SDS-PAGE of FLAG affinity purified mouse HTT (1–549) Q7 protein. Cells from 3 L culture were lysed by freeze/thaw in a buffer containing 50 mM Tris pH 7.4, 500 mM NaCl, 10% glycerol, 1% CHAPS, 1 mM EDTA and Complete EDTA-free protease inhibitors. Soluble fractions following centrifugation were incubated with anti-FLAG M2 affinity gel overnight at 4°C before washing (w1–w3) and eluting with 5×1 ml of 0.4 mg/ml FLAG peptide in 50 mM Tris pH 7.4, 500 mM NaCl, 10% glycerol, 1% CHAPS, 1 mM EDTA (e1–e5). M, molecular weight marker (kDa). (B) SDS-PAGE of Superdex 200 16/60 purified mouse HTT (1–549) Q7 protein. M, molecular weight marker (kDa). Ld, sample loaded onto the column. (C) MonoQ 5/50 chromatogram showing ion exchange separation of mouse HTT (1–549) Q7 protein. The mouse HTT (1–549) Q7 protein eluted as two distinct peaks in the middle of the NaCl gradient. (D) SDS-PAGE of MonoQ 5/50 ion exchange purified mouse HTT (1–549) Q7 protein. M, molecular weight marker (kDa). Ld, sample loaded onto the column. Protein contained in each peak was separately pooled and concentrated. Protein concentration was determined by Bradford assay, in triplicate. (TIF) [file pone.0096854.s009.tif]
