# Supplementary material for: Quantification Assays for Total and Polyglutamine-Expanded Huntingtin Proteins
Source: PLoS One. 2014 May 9;9(5):e96854. doi: 10.1371/journal.pone.0096854 (PMC4016121; doi:10.1371/journal.pone.0096854)
Supplement: Text S1 — (DOCX) [file pone.0096854.s010.docx]

Supplementary data to:

**Quantification Assays for Total and Polyglutamine-expanded Huntingtin Proteins**

**RESULTS**

**Production of human and mouse HTT proteins**

The N-terminal FLAG tagged constructs expressing residues 1-573 (relative to GenBank accession CAD38447.1) of the human HTT protein carrying 23 or 73 glutamines (HTT (1-573) Q23 and HTT (1-573) Q73) were transfected into Sf9 insect cells to generate recombinant baculoviruses. Baculovirus-infected Sf9 cells were harvested and large fragment recombinant HTT proteins purified using anti-FLAG affinity resin. Analysis of the eluted fractions by SDS-PAGE evidenced the presence of additional proteins/ truncated products (Supplementary Figure S7A). Further purification of each recombinant HTT protein was performed using gel filtration chromatography and fractions for both HTT (1-573) Q23 and HTT (1-573) Q73 were pooled and concentrated. After purification, a second band of lower molecular weight was still detected in the HTT (1-573) Q23 sample, as evidenced by SDS-PAGE (Supplementary Figure S7B), which may be a breakdown product of HTT (1-573) Q23 or a different protein that has co-purified with HTT (1-573) Q23.

Polyglutamine expanded HTT protein is susceptible to aggregation [[12](#_ENREF_12),[34](#_ENREF_34)]. Therefore, stability studies of the purified HTT proteins were performed to determine optimal storage conditions. Purified proteins were stored either in a buffer containing 50 mM Tris pH 7.4, 500 mM NaCl, 10% glycerol, 0.1% CHAPS, 1 mM EDTA or with additional glycerol (25% - 50%), BSA (0.25%) or additional sodium chloride. Native gel analysis of protein samples stored at -80°C for two months (57 days) which had undergone five freeze/thaw cycles showed an identical pattern to samples stored at -80°C for 72 h which had undergone one freeze/thaw cycle for all the conditions tested (Supplementary Figure S8A). The lower, more intense band is likely to be folded monomeric protein whereas the higher, less intense band may be aggregated or unfolded protein. After five freeze/thaw cycles no additional bands have appeared and the ratio of the intensity of the lower band to the higher band in each storage condition remains the same, indicating that the monomeric protein has not aggregated or formed higher molecular weight complexes. Native gel analysis of purified HTT (1-573) Q23 and HTT (1-573) Q73 large fragment proteins stored in 50 mM Tris pH 7.4, 500 mM NaCl, 10% glycerol, 0.1% CHAPS, 1 mM EDTA was also carried out after one year of storage (386 days) (Supplementary Figure S8B). The analysis evidenced two extra higher molecular weight bands which were not observed after two months of storage and which are likely to be aggregated forms of HTT.

The N-terminal FLAG tagged construct expressing residues 1-549 (relative to RefSeq accession number NM_010414) of the mouse HTT protein carrying seven glutamines (Htt (1-549) Q7) was transfected into Sf9 insect cells to generate recombinant baculovirus, as already described for human N-terminal HTT fragment constructs [42]. Baculovirus-infected Sf9 cells were harvested and large fragment recombinant mouse HTT protein was purified using anti-FLAG affinity resin. Analysis of the eluted fractions by SDS-PAGE evidenced the presence of additional proteins/ truncated products (Supplementary Figure S9A). Further purification using gel filtration chromatography did not significantly improve the purity of the mouse HTT protein (Supplementary Figure S9B), so an additional purification by ion exchange chromatography was performed. The mouse HTT protein eluted from the ion exchange column as two distinct peaks (Supplementary Figure S9C and S9D) which were separately pooled and concentrated. Since ion exchange chromatography separates proteins based on their charge, the different elution profiles could be due to differently phosphorylated forms of the mouse HTT protein.

**Assessment of expanded polyglutamine human HTT MSD assay power**

Pre-clinical studies should be adequately powered to ensure detection of predicted changes in protein expression levels. Therefore, the required sample group size based on the inter-animal variability was assessed for the expanded polyglutamine human HTT assay. To this aim, we mimicked a progressive decrease of mutant HTT, induced by a hypothetical treatment, by diluting HD mouse brain extracts into increasing amounts of lysates generated from correspondent wild-type control mice.

Whole brain homogenates generated from BAC HD (n=7, 3 month-old), zQ175 KI (n=5, 3 month-old) and R6/2 (n=7, 4 week-old) HD mouse models were mixed at different ratios with a brain extract generated from one correspondent age-matched wild-type control animal. The used ratios (HD:WT) were: 100:0, representing the untreated group expressing 100% mutant HTT; 75:25 and 50:50, representing the treated groups showing progressive decrease of mutant HTT (25% and 50%, respectively). We observed a decrease of the MSD signal with progressively decreasing concentrations of the various HD mouse homogenates added to the samples (Supplementary Figure S6). The signal decrease appeared to be linear and the residual signal and the signal reduction showed values close to the theoretical numbers. Inter-animal biological variability, calculated as coefficient of variation, was found to be below 20% for all the HD mouse models tested (Supplementary Figure S6).

The power of the expanded polyglutamine MSD assay for the different HD mouse model was established and the sample size was determined from mathematical equations (Supplementary Table S2) as described in Materials and Methods.

**TABLES**

**Table S1**

Anti-HTT antibodies

| **Antibody** | **Description** | **Epitope** |
| --- | --- | --- |
| BML-PW0595 | Rabbit polyclonal antibody raised to synthetic peptide corresponding to aa 2-17 | ATLEKLMKAFESLKSFQ |
| CHDI-90000137 (pAb137) | Rabbit polyclonal antibody raised to a synthetic peptide corresponding to aa 4-19 | acetyl-LEKLMKAFESLKSFQQC-amide |
| CHDI-90000145 (pAb145) | Rabbit polyclonal antibody raised to a synthetic peptide corresponding to aa 32-53) | acetyl-QQQQQQQQQQQPPPPPPPPPPP--Ahx-C-amide |
| CHDI-90000146 (pAb146) | Rabbit polyclonal raised to a synthetic peptide corresponding to the proline rich region of human HTT (epitope derived from human protein sequence that is divergent from the mouse protein sequence) (aa 54-70) | acetyl-QLPQPPPQAQPLLPQPQC-amide |
| CHDI-90000147 (pAb147) | Rabbit polyclonal antibody raised to a synthetic peptide corresponding to the proline rich region of mouse HTT (epitope derived from mouse protein sequence that is divergent from the human protein sequence) (aa 37-53 of mouse Htt GenBank accession NP_034544) | acetyl-PPPQPPQPPPQGQPPPPC-amide |
| CHDI-90000148 (pAb148) | Rabbit polyclonal antibody raised to a synthetic peptide corresponding to the C-terminal of Exon 1 region (aa 79-92) | acetyl-CPPGPAVAEEPLHRP-amide |
| MW1 | Monoclonal antibody able to detect the expanded polyQ domain (Ko et al., 2001) | DRPLA-19Q |
| MAB2166 | Monoclonal antibody raised against a fragment (aa 183 to 812 as a fusion protein). | GKVLLGEEEALEDDS  (Cong et al., 2005) |

*Note.* Unless otherwise indicated, amino acid numbers indicate the antigenic peptides on human HTT GenBank accession CAD38447.1 carrying 23 CAG and 7 CCG repeats.

**Table S2**

Expanded polyQ human HTT MSD assay power for various HD mouse models.

| **BAC HD** | | **zQ175 KI** | | **R6/2** | |
| --- | --- | --- | --- | --- | --- |
| Effect size | Group size | Effect size | Group size | Effect size | Group size |
| 25 % | N = 2 | 25 % | N = 3 | 25 % | N = 3 |
| 50 % | N = 2 | 50 % | N = 2 | 50 % | N = 2 |

*Note.* The effect size refers to percentage reduction of mutant human HTT protein levels.
